# Restoration of aged hematopoietic cells by their young counterparts through instructive microvesicles release

**DOI:** 10.18632/aging.203689

**Published:** 2021-11-11

**Authors:** Steven J. Greco, Seda Ayer, Khadidiatou Guiro, Garima Sinha, Robert J. Donnelly, Markos H. El-Far, Lauren S. Sherman, Yannick Kenfack, Sri Harika Pamarthi, Marina Gergues, Oleta A. Sandiford, Michael J. Schonning, Jean-Pierre Etchegaray, Pranela Rameshwar

**Affiliations:** 1 Department of Medicine, Rutgers New Jersey Medical School, Newark, NJ 07103, USA; 2 Rutgers School of Graduate Studies, Rutgers New Jersey Medical School, Newark, NJ 07103, USA; 3 Department of Biological Sciences, Rutgers University, Newark, NJ 07102, USA

**Keywords:** age, hematopoiesis, bone marrow, miRNA, microvesicles

## Abstract

This study addresses the potential to reverse age-associated morbidity by establishing methods to restore the aged hematopoietic system. Parabiotic animal models indicated that young secretome could restore aged tissues, leading us to establish a heterochronic transwell system with aged mobilized peripheral blood (MPB), co-cultured with young MPB or umbilical cord blood (UCB) cells. Functional studies and omics approaches indicate that the miRNA cargo of microvesicles (MVs) restores the aged hematopoietic system. The *in vitro* findings were validated in immune deficient (NSG) mice carrying an aged hematopoietic system, improving aged hallmarks such as increased lymphoid:myeloid ratio, decreased inflammation and cellular senescence. Elevated MYC and E2F pathways, and decreased p53 were key to hematopoietic restoration. These processes require four restorative miRs that target the genes for transcription/differentiation, namely *PAX* and phosphatase *PPMIF*. These miRs when introduced in aged cells were sufficient to restore the aged hematopoietic system in NSG mice. The aged MPBs were the drivers of their own restoration, as evidenced by the changes from distinct baseline miR profiles in MPBs and UCB to comparable expressions after exposure to aged MPBs. Restorative natural killer cells eliminated dormant breast cancer cells *in vivo*, indicating the broad relevance of this cellular paradigm - preventing and reversing age-associated disorders such as clearance of early malignancies and enhanced responses to vaccine and infection.

## INTRODUCTION

Aging is a risk factor for chronic diseases, resulting in high morbidity, decreased quality of life and increased health care cost [1]. Over time, continuous intracellular stress leads to disrupted tissue physiology such as perturbated tissue homeostasis, stem cell exhaustion, and increased cellular senescence [2]. Cumulative age-associated changes could be caused by external and replicative stress that alters the epigenetic dynamics [3]. These changes could predispose cells to oncogenic events, bypassing the default protection [4]. Outcomes of emerging drugs to reverse age underscore the complex aging process and demonstrate the challenge of using a single drug [5].

Age is associated with hematological disorders such as anemia, malignancies, reduced innate immune function and non-hematological disorder, e.g., diabetes and cardiovascular disease [6]. In the aged bone marrow (BM), the relationship between hematopoietic stem cells (HSCs) and their supporting niche cells such as stroma are functionally dysregulated [7, 8]. The aging stromal cells release soluble and vesicular secretome to create an inflammatory milieu, increased cellular senescence and enhanced cell cycle [9, 10]. Increased numbers of HSCs in the aged are functionally impaired displaying features such as defective transplantability, loss of heterogeneity, increased genomic mutations, metabolic switch and myeloid bias [2, 8, 11–15]. Single driver mutation in the aged hematopoietic cells can lead to the emergence of non-malignant clones of indeterminate potential (CHIP) thereby increasing the risk of hematological malignancy [16]. The aged neural system could also influence BM functions, directly by innervation and indirectly by neurohormones [17–19].

The documented defects on hematopoietic aging have not been fully leveraged to reverse and/or halt the aging process [20]. Considering the expanded lifespan of humans, fulfilling this gap would have an impact on global public health and the economy. We propose an efficient and non-invasive therapeutic strategy that functionally restores the hematopoietic system. This method could be applied in preventive therapy for middle age individuals and as a potential treatment for the aged population [12]. Thus, restoring the aged hematopoietic system would improve the body’s immune surveillance to eliminate emerging malignancies, effective response to infections and enhanced functions of other organs [21, 22].

The seminal parabiotic models [23] were subsequently shown to improved cognitive, cardiac and skeletal muscle function of the older animals [24–28]. These parabiotic models as well as others employing partial cellular reprograming did not report on changes in the hematopoietic/immune systems [29]. Several studies indicated that there are precedents for hematopoietic rejuvenation. Young endothelial cells can reverse hematopoietic defects exerted by aged BM endothelial cells [30]. Dedifferentiated aged HSCs into iPSCs led to improved hematopoietic function [31]. Improved HSC function from atomic bomb survivors occurred when they were placed in a competent niche [15, 32]. Cdc42 inhibitor was also reported to restore aged HSC function [33].

We developed a transwell system with aged and young hematopoietic cells that allowed for intercellular communication in a heterochronic system though their secretome. The heterochronic culture system showed restoration of the aged hematopoietic system by young mobilized peripheral blood (MPB) or umbilical cord blood (UCB), independent of allogeneic differences between the two donor cells. MiRNAs within secreted microvesicles (MVs) were responsible for the improved hematopoietic functions, through increased expression of MYC- and E2F- targeted genes, and decreased p53. The restoration paradigm in the heterochronic cultures were corroborated with immune deficient mice (NSG) carrying an aged human hematopoietic system, resulting in improved aging hallmarks - senescence, inflammation and increased lymphoid:myeloid ratio, similar to young humans [8]. Omics approaches identified the restorative miRNAs, which acted by regulating the genes for transcription/differentiation PAX and phosphatase PPMIF [34, 35]. These miRs were sufficient to restore the aged hematopoietic system in the NSG mice. Collectively, the results showed promise for age related disorders. Notably, enhanced natural killer (NK) activity in the restored aged cells were able to target dormant breast cancer cells in mice femurs, supporting the potential in age-related dysfunction such as malignancy [36]. We objectively discuss the implications for the hematopoietic restoration method in medicine.

## RESULTS

### Hematopoietic restoration of aged mobilized peripheral blood cells (MPBs)

MPBs are comprised of mixed cell subsets that include mature and immature hematopoietic cells. Furthermore, although the cellular frequency of particular cell subsets are different between young and aged MPBs, the compositions are similar. Thus, we evaluated these two sources of MPBs for baseline hematopoietic cells in clonogenic assays for CFU-GM (colony forming units-granulocytic monocytic) and BFU-E (burst forming units-early erythroid). There were similar numbers of CFU-GM between the two groups, but significantly (*p* < 0.05) less BFU-E in young MPBs (Supplementary Figure 1A). Flow cytometric analyses indicated comparable frequency of CD34+/CD38- cells (primitive hematopoietic cells) in the young and aged MPBs, but significantly (*p* < 0.05) more CD34+/CD38+ (progenitors) in young MPBs (Supplementary Figure 1B).

**Figure 1:**
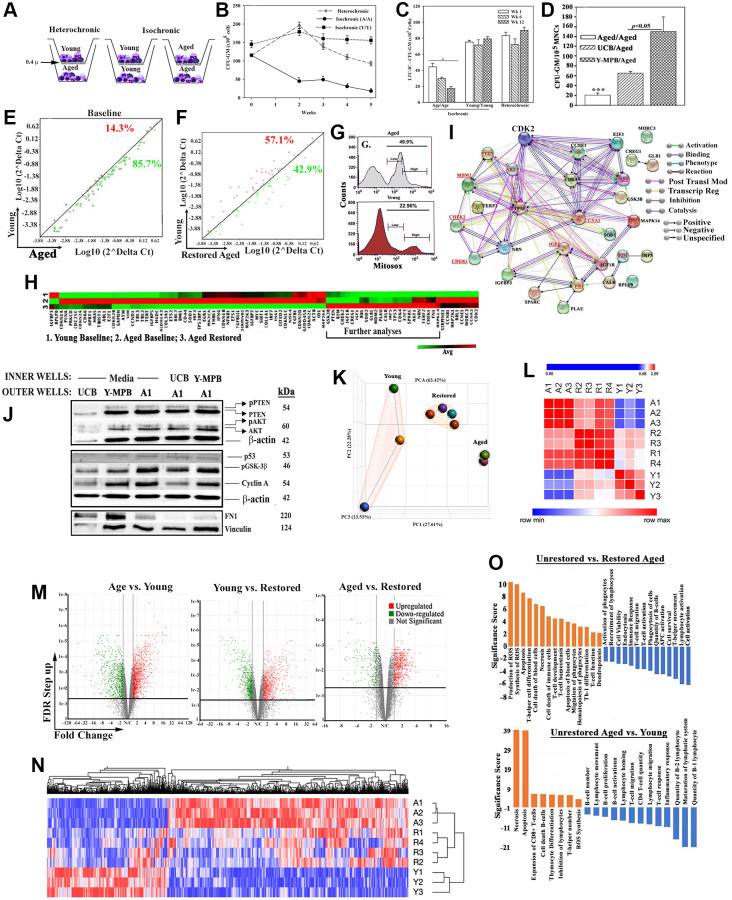
***In vitro* hematopoietic restoration of aged MPBs.** (**A**) Cartoon shows the method employed for non-contact isochronic and heterochronic cultures. (**B**) Timeline clonogenic assays for CFU-GM with viable cells from isochronic (aged or young MPBs) and heterochronic cultures (restored aged MPBs). The results are presented as mean CFU-GM ± SD (*n* = 8 donors, each donor tested in triplicate with two young donors). ^*^p < 0.05 vs. similar time points in heterochronic cultures; ^**^p < 0.05 vs. time 0 and wks 2, 3 and 4. (**C**) LTC-IC cultures were established using the model in ‘A’ except for seeding the aged MPBs on confluent γ-irradiated BM stromal cells in the outer wells. Control cultures contained isochronic young or aged cells in both wells. At wks 1, 6 and 12, clonogenic assay for CFU-GM with aliquots of viable mononuclear cells. The values for each time point were plotted together (8 donors, each tested in triplicates, CFU-GM/10^5^ MPBs ± SD. ^*^p < 0.05 vs. heterochronic. (**D**) Heterochronic and isochronic cultures were established with 10^7^ UCB in the inner wells. At wk 4, aliquots of aged MPBs were analyzed for CFU-GM and the results presented as mean CFU-GM ± SD for 5 different UCB, each tested in duplicate. ^***^p < 0.05 vs. heterochronic cultures with UCB. (**E**) Senescence-related gene expression was performed with 84-gene qPCR arrays using cDNA from restored and unrestored (baseline) aged and young MPBs. Gene expression for 4 donors was determined by calculating the ΔC_t_ between gene-of-interest and housekeeping genes and then plotted as Log_10_(2^ΔCt^). Each dot represents the average gene expression for donors. Baseline comparison for unrestored young vs. aged MPBs is shown in red for higher expression in young and green for higher expression in aged. The line *y = x* indicates no change. (**F**) The analyses described in `E’ was performed for young and restored and the data are similarly presented. (**G**) Oxidative stress by MitoSox assay, delineated as MitoSox, negative, low and high by flow cytometry. (**H**) Hierarchical clustering with the array data from ‘E and F’. (**I**) The genes upregulated in the qPCR array in ‘H’ (open boxed region) were analyzed by RAIN to demonstrate predicted interactions. (**J**) Western blot (3 biological replicates) with whole cell extracts from unrestored young MPB (Y), UCB and restored A1 (restored with UCB or Y-MPB). SDS-PAGE: top, 15%; middle 12%; bottom, 6%. (**K**) PCA of RNA-Seq data from MPB (3 young, 3 age) and 4 restored MPBs. Lines highlight the groups. (**L**) Similarity matrix of ‘A’ for young, age and restored samples. (**M**) Volcano plot of differentially expressed genes. (**N**) Heatmap of fold changes with an FDR ≤ 0.05 as a cut off with linked significant pathways. (**O**) IPA-determined significant hematological functions with shown comparisons. See also Supplementary Table 1, Supplementary Figures 1 and 2.

The hematopoietic effects by young MPBs on aged MPBs were studied in a transwell system in which the inner and outer wells contained equal amounts (10^7^) of young and aged MPBs, respectively (heterochronic culture) (Figure 1A). Control isochronic cultures contained aged or young MPBs in both wells. The 0.4 μm transwell membrane allowed for the passage of soluble factors and microvesicles (MVs), but prevented cell transfer [37] (Supplementary Figure 1C). The initial studies conducted a time-course investigation up to wk 5 using clonogenic assays for CFU-GM with the aged cells as readouts of hematopoietic activity. CFU-GM progenitors are excellent representation of hematopoietic activity in the samples. Controls included isochronic cultures with aged or young MPBs in both inner and outer wells. There was significantly (*p* < 0.05) more CFU-GMs with heterochronic cultures as compared to isochronic aged cultures but similar numbers (*p* > 0.05) with isochronic young cultures (Figure 1B). We also evaluated the phenotype of the restored aged cells and observed mature immune cells such as CD3 and NK cells (Supplementary Figures 1 and 6). These timeline studies combined with the phenotype shown at wk 7 led us to select the 7-day time point for subsequent studies.

The enhanced activity by hematopoietic progenitors (Figure 1B) led us to ask if similar outcome occurred at the level of HSCs. We performed long-term culture-initiating cell (LTC-IC) assay in the outer wells of heterochronic cultures since this assay is an *in vitro* surrogate of HSC function [38]. Control isochronic cultures included young or aged cells in the inner and outer wells. At wks 1, 6 and 12, CFU-GMs in the outer wells of heterochronic cultures (*n* = 8, each in triplicate) were significantly (*p* < 0.05) increased, relative to isochronic aged cultures but were similar to isochronic young cultures (Figure 1C). Since CFU-GMs at wk 12 are derived from the seeded HSCs, their increase indicated increase in LTC-IC/HSC function with young cells.

### Hematopoietic restoration - independent of donor allogenicity

We determined if allogeneic differences between the age and young donors influenced hematopoietic activity in the heterochronic cultures. Such possibility could occur by MHC-II transfer from the young cells to activate the immune cells within the aged cells [39, 40] (Supplementary Figure 1D). We addressed this question by asking if the restored aged cells stimulated their naïve counterparts (unrestored autologous cells). One-way mixed lymphocyte reaction (MLR) with restore aged MPBs as stimulators and unrestored/freshly thawed aged autologous MPBs as responders, showed baseline stimulation, indicating no allogeneic influence by the young cells (Supplementary Figure 1E).

Next, we asked if MHC-II density on young cells could influence the restoration process. We used umbilical cord blood (UCB) as the source of young cells due to relatively higher MHC-II on their HSCs, which we corroborated in our samples [41] (Supplementary Figure 1F). Parallel heterochronic cultures with UCB or young MPBs and the same aged cells indicated significantly (*p* < 0.05) more CFU-GM with UCB, relative to isochronic aged cultures, but significantly (*p* < 0.05) less than young MPBs (Figure 1D). One-way MLR with UCB showed no allogeneic response (Supplementary Figure 1G).

MVs can transfer MHC-II leading us to examine these MVs for MHC-II [37, 42]. Flow cytometry for MHC-II on heterochronic MVs indicated background fluorescence whereas the positive control (activated peripheral blood mononuclear cells) showed bright fluorescence (Supplementary Figure 1H and 1I). Together, the data indicated that allogeneic difference between the young and aged cells did not contribute to aged cell restoration.

### Improved senescence of aged cells in heterochronic cultures

Cellular senescence is an established aging hallmark, which prompted us to ask if the heterochronic cultures improved the senescence gene profile of these cells [10, 43]. Senescence gene arrays (84-PCR), using a 4-fold cutoff, indicated 85% increase for unrestored/baseline aged cells and 43% for restored cells (Figure 1E, 1F, Supplementary Figure 2A and 2B). The values were similar with variations for each time point, ±10^3^. Since the purpose of the studies was to evaluate the changes in senescence genes, we plotted the averages in the scatter plot. Similar analyses with senescence secretome arrays (SASP, 68 antibodies) identified 13 proteins in the media of baseline aged cells and 4 with in the media of heterochronic/restored cultures [44] (Supplementary Figure 2C–2G). Collectively, the restoration paradigm improved the senescence gene profile of aged MPBs.

### Molecular changes - amenable to balanced hematopoietic activity

The vast difference in oxidative stress between young and aged samples (Figure 1G) led us to further analyze the senescence PCR data. Hierarchical clustering using the averages from biological replicates indicated striking similarities between young and restored MPBs (Figure 1H). A cluster of 30 expressed genes (Boxed region), when subjected to RNA-protein analysis (RAIN), showed a gene network comprised of hematopoietic regulatory programs (Figure 1I, Supplementary Figure 2H). Including is MDM2 expression that can decrease p53, which is consistent with improved senescence profile in the restored samples. Decreased p53 would not compromise DNA repair due to CHEK1/2 within the network [45]. Upregulated IGF1, which is associated with longevity, was increased in the restored cells [46]. Decreased fibronectin (FN1) in the restored cells could improve risk for hematopoietic pathology such as myelofibrosis [47].

We validated key proteins within the network (Figure 1I-underline) by Western blot using extracts from aged cells (Figure 1J, densitometric analyses, Supplementary Figure 2I). Increases in p-PTEN and p-AKT in the restored cells are amenable to balanced cell proliferation; increased cyclin A can decrease G2 transition to maintain hematopoietic cell quiescence; higher GSK-3β suggested a pathway involving β-catenin to benefit hematopoietic function. Consistent with increased MDM2 in the PCR array was decreased p53 protein (Supplementary Figure 2I). In summary, hematopoietic restoration is associated with decreased senescence and increased expression of genes to benefit hematopoietic activity.

### Molecular landscape in unrestored versus age restored MPBs

We examined global changes with RNA-Seq by comparing the following: restored and unrestored age cells, and young MPBs. Principle component analyses (PCA) showed distinct clustering among the groups (Figure 1K). Although one young donor was not within the cluster of the other two young donors, similarity matrix showed the restored samples moving closer to the young profile (Figure 1L). Applying a false discovery rate (FDR) cutoff of <0.05, we selected 2,140 genes, visualized in volcano plots and hierarchical clustering (Figure 1M and 1N). Since the hierarchical plot was established with >2,000 genes, we conducted additional analyses to understand the molecular changes with the restored aged MPBs.

**Figure 2:**
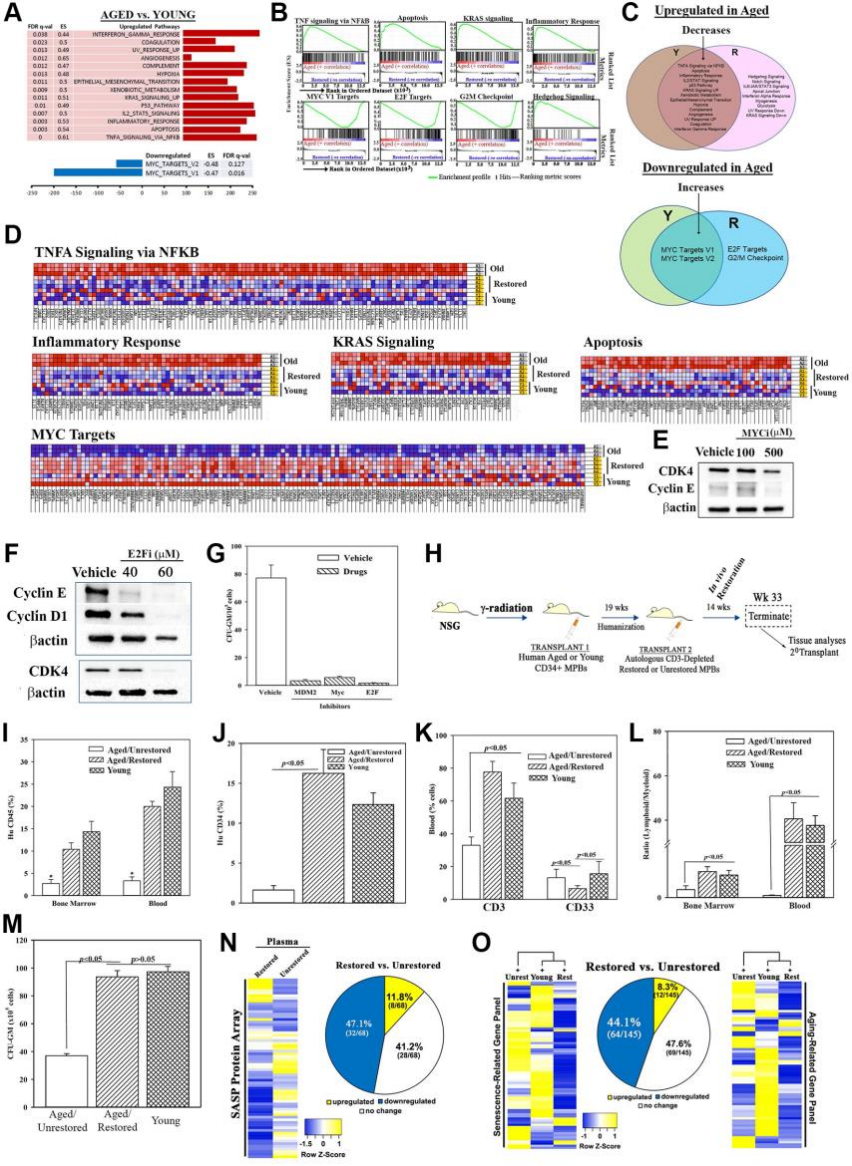
**Molecular changes in age-related pathways following restoration.** (**A**) Up- and down-regulated pathways in age, relative to young MPBs. (**B**) Enrichment plots for the top and down-regulated pathways. (**C**) Venn diagram shows shared and unique pathways between young and restored group, and overlap, changed pathways with restoration. (**D**) Enriched heatmaps of significant changes in ‘G’ (Full heat maps Supplementary Figure 2J). (**E**, **F**) Western blot for cell cycle proteins with extracts from 3 restored cells, MYC or E2F inhibitors or vehicle. (**G**) Clonogenic assay for CFU-GM with cells restored with 1 μM MDM2, 100 μM MYC or 40 μM E2F, mean ± SD (4 different aged donors, restored with 2 young donors; each in triplicates). See also Supplementary Figure 2). (**H**) Overview of NSG transplanted with aged huCD34+ MPBs. At the achievement of chimera, NSG with an aged hematopoietic system are injected with autologous restored (*n* = 12) or unrestored (*n* = 11) CD3-depleted MPBs. Serial transplantation used wk 33 huCD34+ cells. Controls were given young CD34+ cells (*n* = 8). (**I**–**K**) Flow cytometry for huCD45+ cells in BM and blood, ^*^p < 0.05 vs. the other groups, (**I**), huCD34+ cells in BM (**J**), huCD3+ and huCD33+ cells in blood (**K**), mean % cells ± SD. (**L**) Lymphoid (CD3^+^+ CD19^+^)/myeloid (CD33^+^) ratio in BM and blood. (**M**) CFU-GM in cultures with huGM-CSF and huIL-3 and huCD45+ cells from BM, mean ± SD. (**N**) SASF array with huNSG plasma. Semi-quantitative densitometry used 1.5-fold cutoff for classification as up- or down-regulated, or no change. Heatmaps and piechart for differential gene expression. (**O**) RNA from huCD45+ BM cells evaluated qPCR gene arrays. Normalization to housekeeping genes used 1.5-fold cutoff, mean ± SEM. ^*^p ≤ 0.05 vs. control. See also Supplementary Figures 2 and 3.

Ingenuity pathway analyses (IPA) identified top pathways associated with hematopoietic development and, decreases in reactive oxygen species, apoptosis and necrosis (Figure 1O). Gene Set Enrichment Analysis (GSEA) identified significantly up- and down-regulated (FDR *q* value < 0.05) pathways in age and young groups (Figure 2A and 2B). MYC pathways were the only downregulated targets in the age group with 14 upregulated pathways. Restoration improved key age-related functions - inflammation, immune suppression and cell death - TNFα signaling, NFκB signaling, apoptosis and p53. The Venn diagram depicts the pathways corrected by restoration along with the shared and unique pathways (Figure 2C). The up- and down-regulated pathways in the aged samples transitioned into the overlapping section with young following restoration. Heatmaps are shown for the enriched sections of the top shared pathways (Figure 2D and Supplementary Figure 2J).

### MYC, E2F and p53 in hematopoietic restoration

This section validated key pathways identified in the RNA-Seq analyses. MYC pathway, which was increased with restoration could explain hematopoietic restoration by partial reprogramming [29, 48]. E2F, due to its role in cell cycle regulation, which is important for hematopoietic homeostasis. We selected p53 because of increased MDM2 in the restored cells (Figure 1I, 1J and 1O). Reduced p53 would mitigate cellular senescence. Heterochronic cultures, in the presence or absence of MYC, E2F or MDM2 inhibitors or vehicle, indicated decreases in proteins linked to G1 transition by Western blot (Figure 2E and 2F). Clonogenic assay for CFU-GM in the cultures showed significant (*p* < 0.05) decreases with the inhibitors relative to vehicle (Figure 2G). In summary, decreased p53, and increases in MYC and E2F pathways are important for restoring the function of aged MPBs.

### *In vivo* restoration of the aged hematopoietic system – humanized NSG (huNSG)

This section recapitulated the *in vitro* restoration in huNSG mice carrying aged hematopoietic system. We achieved hematopoietic chimera in NSG mice by transplanting aged CD34+ cells using dose-response and time-course studies. Mice began to achieve chimera after 8 weeks, based on human CD45+ cells in mice blood (Supplementary Figure 3A and 3B, depicted baseline chimerism prior to the second transplant). Mice with stable chimera (wk 19) were transplanted with CD3-depleted restored MPBs. Mice remained healthy until the end-point (Figure 2H, Supplementary Figure 3C–3H). After wk 14, we analyzed the blood, spleen and BM of mice for immune cells by flow cytometry (Supplementary Figure 3I–3N). Mice transplanted with restored cells showed significantly (*p* < 0.05) more huCD3 and huCD34+ cells, decreased myeloid cells and increased lymphoid:myeloid ratio, as compared to mice given unrestored aged cells (Figure 2I–2L). Clonogenic assays for CFU-GM using huCD45+ cells from mice femurs indicated a significant (*p* < 0.05) increase when mice were given restored cells as compared to those given unrestored aged cells (Figure 2M). These improvements correlated with enhancement in senescence profile: plasma, 12% restored vs. unrestored cells; qPCR senescence/aging array with femur cells, 8%, restored cells vs. unrestored cells (Figure 2N and 2O, Supplementary Figure 3O–3Q).

**Figure 3:**
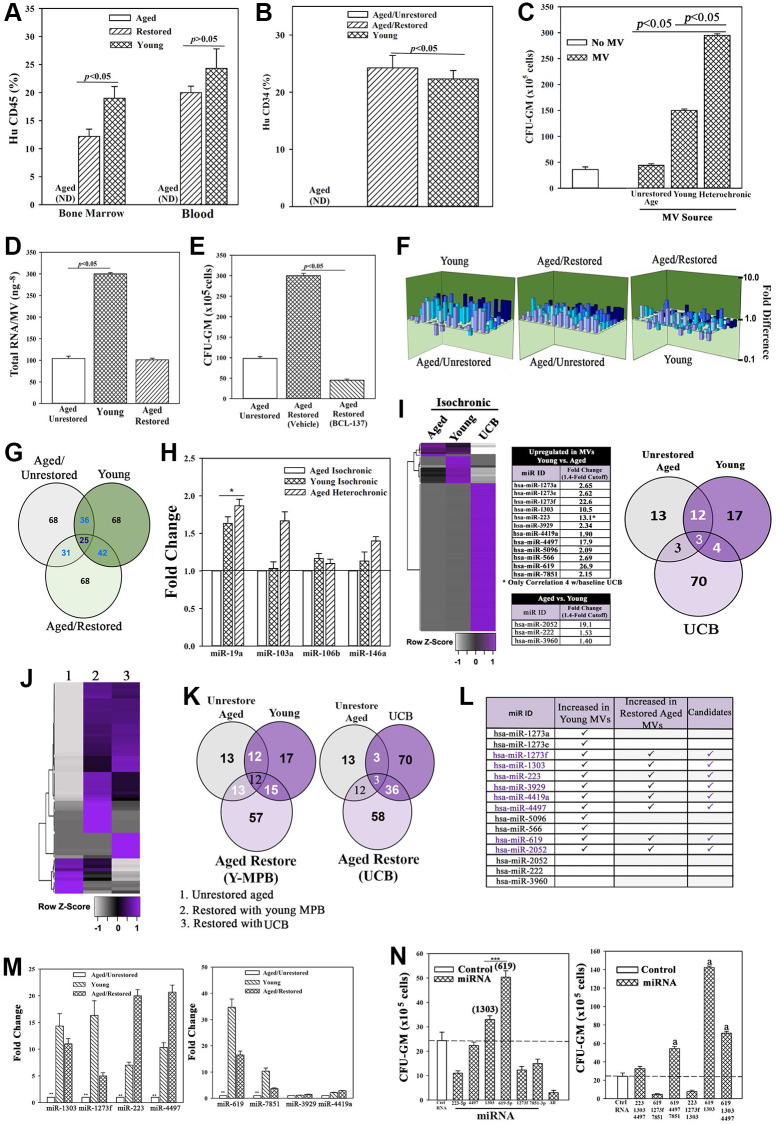
**Restored cells transplanted in NSG mice with aged human hematopoietic system (huNSG) and exosomal RNA in restoration.** (**A** and **B**) HuCD34+ cells (10^5^), pooled from wk 33 mice (Figure 2H) were injected into naïve NSG mice (*n* = 3). At 12 wks, mice were analyzed for huCD45+ and huCD34+ cells. ND = none detected. (**C**) Pooled MVs (10^6^) from heterochronic or isochronic (young and aged) cultures were added to naïve aged MPBs on day 0 and 4 and at day 7, CFU-GMs were assessed in clonogenic assays, mean ± SD, *n* = 5. (**D**) Total RNA in MVs isolated from heterochronic cultures, ng^-8^/MV ± SD, *n* = 8. (**E**) BCI-137 or vehicle was added to heterochronic or isochronic cultures, CFU-GM ± SD, *n* = 5. (**F**) 3D plots with data from qPCR miR array data using RNA from MVs. (**G**) Venn diagram showing differential and overlapping miRNAs. (**H**) qPCR for differentially expressed MV miRs. Shown are the consistently upregulated miR in young isochronic (dark green bar) and heterochronic cultures (striped bar), mean ± SD, *n* = 3. Aged isochronic cultures were assigned a value of 1. ^*^p ≤ 0.05 vs. control. (**I**) MiRnome sequencing used small RNA from MVs of aged and young MPBs or UCB. Heatmaps used miRNA, > 1.4-fold between aged and young samples. Venn diagrams depicts the differential and overlapping miRNAs. (**J**, **K**) Studies, similar to `I’, compared miRNAs, sequenced from MVs of aged isochronic and heterochronic (cultured with young MPB or UCB) samples. (**L**) The 12 miRs showing differential expression between aged and young in ‘I’ were compared to miRs that were increased in heterochronic vs. aged isochronic cultures (**I**, **J**). Shown are the increased 8 miRs in restored cells. (**M**) qPCR for the 8 miRs, 7 biological replicates, each in triplicate. The data are normalized to miR-7641-2 and presented as fold change using 1 for aged control. (**N**) 6 validated miRs or control miRs were expressed, alone (left) or together (right) in 5 biological replicates, each in triplicate. CFU-GM at day 7, mean ± SD. ^*^p ≤ 0.05 vs. control. See also Supplementary Figures 3 and 4.

We assessed the functional competence of HSCs by selecting CD34+ cells from the experimental mice and transplanting into naïve NSG mice. CD34+ cells from mice with restored cells reconstituted NSG mice at week 8 whereas similar transplant with CD34+ cells from mice given aged cells did not achieve chimera (Figure 3A and 3B). In summary, transplantation of *in vitro* restored cells improved hematopoietic function in NSG mice carrying an autologous aged hematopoietic system.

### Microvesicles (MVs) in aged hematopoietic restoration

Secreted MVs with their RNA, protein and lipid cargo could cross the culture membrane to establish communication between the aged and young cells [37, 49]. Also, MVs can regulate hematopoiesis [50]. We therefore determined if MVs, in particular exosomes, could be responsible for restoration of aged MPBs. We characterized the MVs from heterochronic and isochronic cultures and found similar size (Supplementary Figure 4A). Notably, aged MPBs produced higher number of MVs as compared to those released from heterochronic cultures or young MPBs (Supplementary Figure 4B). We also validated the incorporation of MVs into aged cells by added those from day 3 heterochronic cultures to naïve aged cells. The MVs, labeled with CMAC blue dye, were examined for transfer into the aged cells by 2D (EVOS imaging) and 3D (confocal microscopy) imaging (Supplementary Figure 4C; Supplementary Videos 1 and 2).

We tested the role of MVs in aged hematopoietic restoration by pooling those from day 4 and 7 heterochronic cultures. The pooled MVs (10^6^ particles, based on dose response studies) were added to naïve aged MPBs at seeding (time 0) and after 4 days. After 7 days, clonogenic assay for CFU-GMs indicated significantly (*p* < 0.05) more colonies with MVs from heterochronic cultures, relative to those from isochronic young and aged cultures, solidifying a role for MVs in hematopoietic restoration (Figure 3C).

We sought the candidate MV cargo by assessing the RNA content, which was higher in young MPBs as compared to aged MPBs (Figure 3D). We focused on miRNAs due to their role in hematopoiesis [51]. The pharmacological agent, BCI-137 (AGO2 inhibitor), blunted miRNA packaging during MV biogenesis without compromising their release (Supplementary Figure 4D, 4E). BCI-137 reduced the small RNA contents of MVs, including significant (*p* < 0.05) decrease of miRs, and decreased CFU-GM in the heterochronic cultures (Supplementary Figures 4F, 4G and 3E, 3F). These findings indicated a critical role for the miRNA content of MV in aged hematopoietic restoration.

We sought the causative miRNA(s) using an 84-probe array. MVs from isochronic (young and aged) and heterochronic co-cultures showed distinct miR profiles with 25 shared among the groups (Figure 3F and 3G). IPA predicted targets for the differentially expressed miRs and indicated a functional network in which young miRs are poised to prevent aging disorders such as cancer and hematological disorders (Supplementary Figure 4H). MiR-19a, -103a, -106b and -146a were upregulated in the young and restored MVs. Since qPCR only validated miR-19a in the young and restored cells (Figure 3H), we conducted further analyses by RNA-Seq of small RNA within the MVs. Using an expression cut-off of 100 mappable reads, we identified 13 and 17 unique miRs in aged and young MVs, respectively (Figure 3I). 12/17 miRs were higher in young vs. aged, while 3/17 were lower (Figure 3I, middle). The miRnome of MVs from UCB was vastly different with 70 miRs in >100 mapped reads but only 4 shared with young MPBs (Supplementary Figure 4I). Similarly, there were differences in intracellular miRnomes among aged, young, and UCB (Supplementary Figure 4J). Importantly, after restoration with young MPBs or UCB cells, there were common miR profiles in the heterochronic cultures, indicating that aged cells could be driving the MV cargo secreted from young cells (Figure 3J–3K, Supplementary Figure 4K–4O).

Among the 12 differentially expressed miRs between baseline young and aged MPBs, 8 were upregulated in the restored cells (Figure 3I and 3L). We validated the 12 miRs by qPCR, which were normalized with miR-7641-2 due to equal expression among the groups. These studies verified 6/12 miRs (Figure 3L, middle/M, Supplementary Figure 4M–4O). We then asked if the restored cells release MVs containing the validated miRs. To address this, we washed and then transferred the restored aged cells to the inner wells as facilitator young cells in heterochronic cultures (Supplementary Figure 4P). The MVs in these heterochronic cultures indicated the continued presence of miR-223 and -619 (Supplementary Figure 4P).

We performed cause-effect studies by expressing ≥1 of the 6 miRs in aged MPBs. These transfected cells were placed in cultures for 7 days, similar to the transwell cultures. Specifically, we cultured the aged cells to mimic restoration with young MPBs, except that these cultures dissected the restorative effects of specific miRNAs. After the culture period, we performed clonogenic assays for CFU-GM and noted significant (*p* < 0.05) increase in CFU-GM with miR-619 and/or -1303, and in combination with the other miRs (Figure 3N). Thus, miR-619, -1303 and -4497 became the restorative candidates for subsequent studies.

### Downstream targets of restorative miRNAs

**Figure 4:**
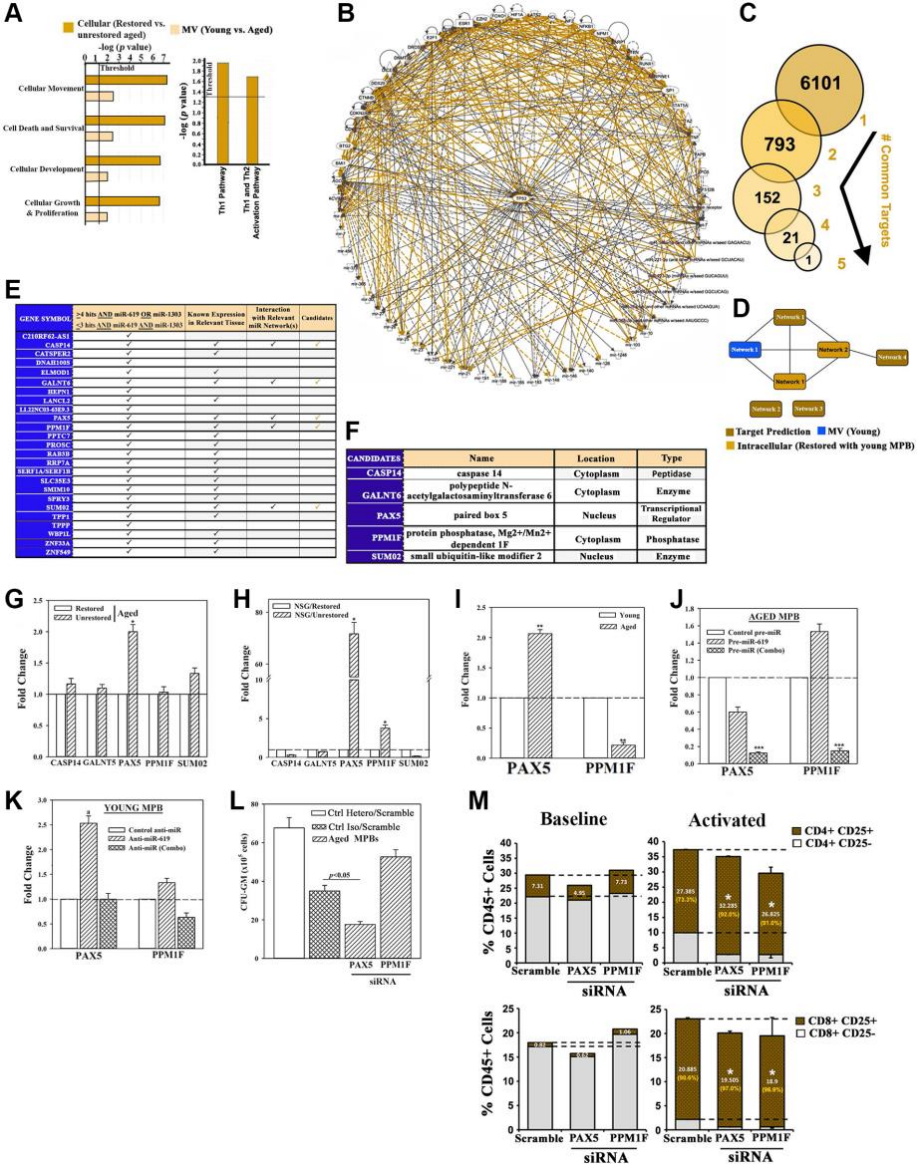
**Exosomal miRNA targets in restoration.** (**A**) IPA output of top predicted cellular functions (left) and canonical pathways (right) in analyses of MV miRNAs from the following: young vs. aged cells, heterochronic vs. aged isochronic cultures. (**B**) Radial depiction of young MV vs. restored intracellular interactome with p53 at the center of overlapping networks (Orange, direct interactions). (**C**) Analyses of 6 miRNAs (Figure 4L) for targets using TargetScan human database. (**D**) Targets were analyzed by IPA and the predicted networks (brown) compared to the young exosomal (blue) and aged heterochronic intracellular (dark orange) miRNA interactome. (**E**) Tabulation of selected targets and predicted interaction with the miRNA interactome. (**F**) 5 potential downstream targets for functional validation. (**G**, **H**) qPCR for candidate targets using RNA from aged cells of heterochronic or isochronic cultures (**G**), or human cells from femurs of huNSG (**H**), Fold change of normalized (β-actin) results, *n* = 4. (**I**) qPCR for PAX5 and PPM1F in aged and young MPBs, fold change with young donor assigned 1. (**J**, **K**) Aged MPBs were transfected with pre-miRs or control miR (**J**) and young MPBs, with anti-miRs or control miR (**K**). At day 7, the cells were analyzed for PAX5 and PPM1F mRNA by qPCR. The data are presented as the mean ± SD fold change, *n* = 4. The controls were assigned values of 1. (**L**) Aged MPBs were transfected with PAX5 or PPM1F siRNA or scramble (control). At day 7, the cells were analyzed for CFU-GM. Positive ctrl: heterochronic cultures, mean CFU-GM ± SD, *n* = 4. (**M**) Effect of PAX5 or PPM1F knockdown by siRNA on T-cell activation (CD25) for CD4^+^ (top panels) and CD8^+^ (bottom panels) populations. Right panels represent the % activated vs. total T-cells shown in orange. See also Supplementary Figure 4.

In order to understand how the MV-containing restorative miRs regulate hematopoietic restoration, we mapped the downstream effector pathways. First, we determined how miRs within the young MVs, and restored (heterochronic) or unrestored (isochronic) aged cells interact to cause functional changes. The miR interactome identified the top cellular pathways, including T-helper 1 and 2, cellular development and molecules that regulate senescence, e.g., CDKN2A and p53 (Figure 4A, Supplementary Figure 4Q and 4R). The greatest convergence between the two datasets was p53 (Figure 4B, orange lines). This outcome is in line with our studies showing p53 with a major role in hematopoietic restoration (Figures 1 and 2). We observed similar findings with the dataset from MVs released from heterochronic cultures using UCB (Supplementary Figure 4S–4V).

We applied miRNA target prediction software for direct targets of the 6 restorative miRNAs. Predicted targets (6101) for the individual miRNAs were stratified for common targets if they include the 6 miRs (Figure 4C). After this, we narrowed the targets if they share >3 miR hits and these hits must include the restorative miR-619 and -1303 (Figure 3). The remaining targets were scanned for known expression in relevant tissues. We eliminated targets encoding hypothetical proteins and if their expression was restricted to neural tissues. We then subjected the targets to predicted pathway analysis (Figure 4D, brown) in the context of our effector-target interactome (Figure 4D - blue and light orange, Figure 4E). The resulting 5 target candidates included the transcription/differentiation PAX5, which was increased in the unrestored isochronic cells, but significantly (*p* < 0.05) decrease after restoration (Figure 4F and 4G) [34]. Both PAX5 and phosphatase PPM1F, which is linked to integrin-mediated adhesion, were decreased in the femurs of restored mice (Figure 4H) [35]. These results led us to examine baseline expression of PAX5 and PPMIF. The results indicated consistent increase in PAX5 in the aged cells, but decrease in PPM1F, perhaps due to its complex function (Figure 4I).

We performed cause-effect studies between the restorative miRs and PAX5/PPM1F expression by transfecting the aged MPBs with pre-miR-619 or pre-1303 and -4497 (combo) or miR mimic (control). Analyses for PAX5 and PPM1F mRNA by qPCR indicated that miR-combo significantly (*p* < 0.05) decreased PAX5 and PPM1F mRNAs, compared to control or pre-miR-619 alone (Figure 4J). In corollary studies, we transfected young MPBs with anti-miR-619 or anti-miR (Combo) and observed increases in both PAX5 and PPM1F with miR-619 (Figure 4K).

Functional studies used aged MPBs, transfected with PAX5 or PPMIF siRNA or scramble siRNA followed by clonogenic assay for CFU-GM. PAX5 siRNA caused a significant (*p* < 0.05) decrease in CFU-GM whereas PPM1F siRNA significantly (*p* < 0.05) increased CFU-GM (Figure 4L). Positive control with heterochronic cultures resulted in the expected colonies. Knockdown of PAX5 and PPM1F led to T-cell activation, which is in line with improved lymphoid functions during restoration (Figure 4M).

### *In vivo* hematopoietic restoration by candidate miRNAs

**Figure 5:**
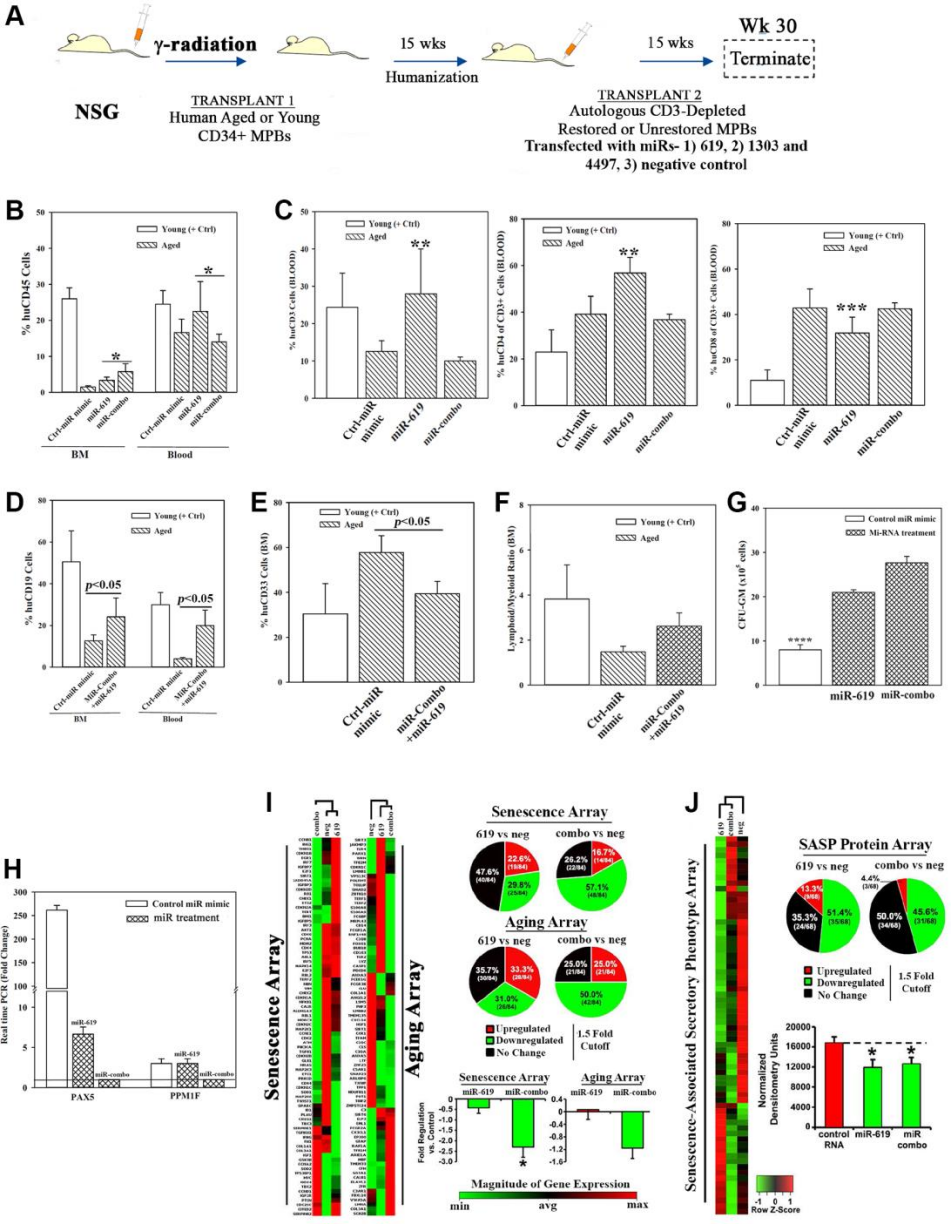
**Hematopoietic restoration with miRNAs.** (**A**) Restoration protocol with NSG was similar to Figure 2H. Chimeric mice were given autologous CD3-depleted aged MPBs, transfected with miR-619, miR-combo, −619, −1303 and −4497, or control (RNA mimic) and then cultured for 7 days, (*n* = 18). (**B**–**E**) Mice were analyzed for huCD45 in BM and blood (**B**); huCD3, CD4 and CD8 in blood (**C**); HuCD19 in blood and BM (**D**); HuCD33 in BM (**E**). Results presented as % mean cells ± SD (**F**) Lymphoid:Myeloid ratio (CD3^+^+CD19^+^/CD33^+^) in BM. (**G**) CFU-GM with huCD45+ cells from femurs, mean ± SD. (**H**) qPCR for PAX5 and PPM1F with RNA from huCD45+cells from femurs. Fold change ± SD used 1 for the lowest value. (**I**, **J**) RNA from `H’ were analyzed in qPCR human senescence and aging arrays. Normalized results used 1.5-fold cutoff to classify up- or down-regulation, or no change (**I**). SASF analyses with plasma. Semi-quantitative densitometry used 1.5-fold cutoff, similar to I (**J**). Differential gene and protein expressions as heatmaps (left), pie charts (top) and bar graph (bottom), mean ± SD. ^*^p ≤ 0.05 vs. control. See also Supplementary Figure 5.

We asked if the restorative miRs (-619 and/or −1303 + −4497 (combo)) (Figure 3) could replace cellular *in vitro* restoration in heterochronic cultures. We addressed this by transplanting aged MPBs, transfected with the restorative miRs in NSG mice carrying aged hematopoietic system (Figure 5A). We selected mice with >1% blood chimera (huCD45+) to transplant aged MPBs that were transfected with miR-combo, miR-619 or control miR mimic (Supplementary Figure 5A and 5B). The mice showed normal pathology (Supplementary Figure 5C–5H). Compare to control miR, there was significantly (*p* > 0.05) more huCD45+ in the BM of mice with cells transfected with the restorative miRs (Figure 5B). Relative to control miR, miR-619 transfectants resulted in significantly (*p* < 0.05) more CD3+ and CD4+ cells and significant (*p* < 0.5) decrease of CD8+ cells (Figure 5C). The lymphoid:myeloid ratio, which is a hallmark of reverse aging was increased, with significant (*p* < 0.05) increase of CD19+ cells and decreased (*p* < 0.05) CD33+ myeloid cells (Figure 5D–5F).

Clonogenic assay for CFU-GM using huCD45+ cells from mice femur indicated significant (*p* < 0.05) increase when mice were given miR-combo- or miR-619-transfected aged cells as compared to the group given control miR mimic (Figure 5G). PAX5 level was significantly (*p* < 0.05) decrease in mice with miR-619 or miR-combo transfectants (Figure 5H). Similar decrease was observed for PPM1F, but only for miR-combo (Figure 5H).

The miR-mediated restoration also reduced cellular senescence, based on PCR array with cDNA from huCD45+ femur cells (Figure 5I, Supplementary Figure 5I–5K). MiR-combo significantly (*p* < 0.05) decreased (>50%) the senescence/age-related genes. SASP factors in mice plasma showed 51.4% and 45.6% decreases for miR-619 and miR-combo, respectively (Figure 5J). Taken together, the miRs restored hematopoiesis similar to cells from heterochronic co-cultures, and highlighted roles for the transcription factors PAX5 and PPM1F in restoration.

### Enhanced Natural killer (NK) activity within restored old MPB in immune therapy

**Figure 6:**
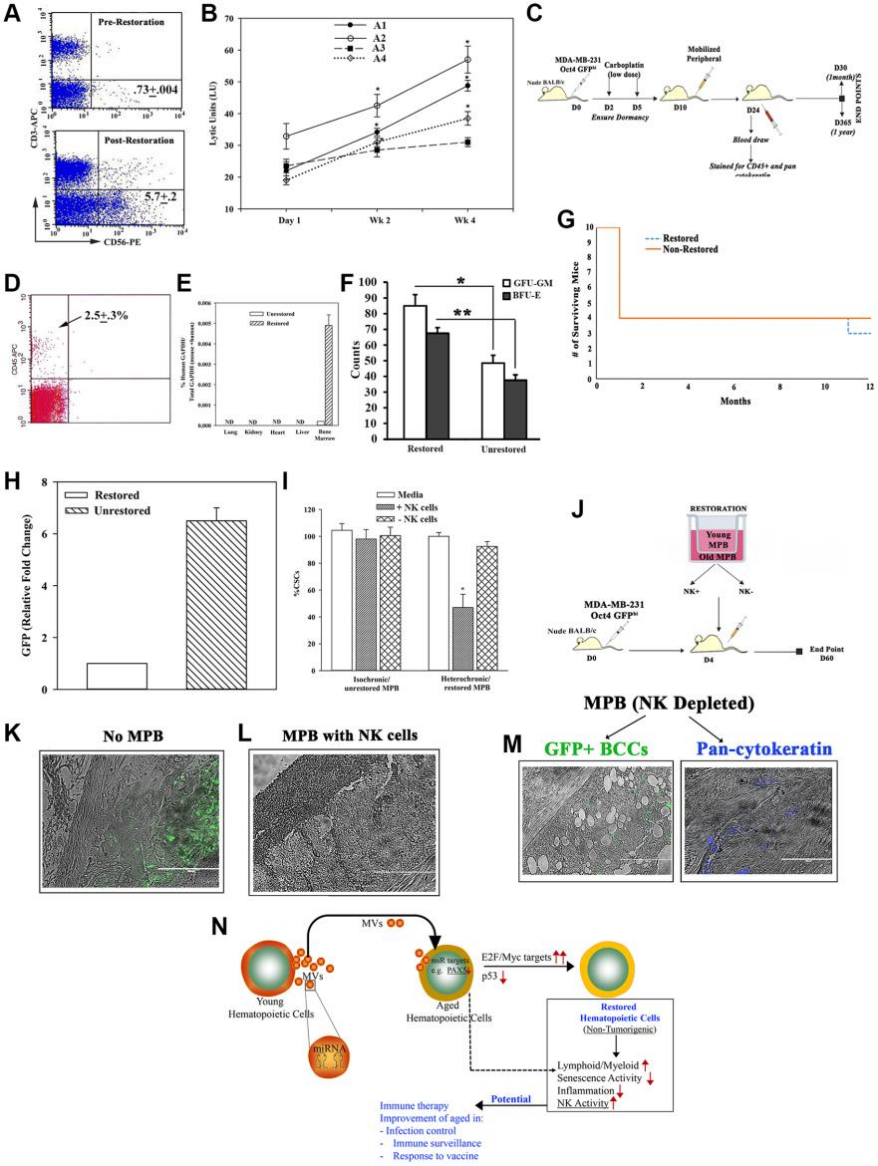
**Restorative containing NK cell function.** (**A**) Flow cytometry for CD56+ cells, pre- and post-restoration. (**B**) Timeline LUs (Supplementary Figure 6A for calculation) in restored cells. (**C**) Treatment protocol with mice harboring dormant CSCs. (**D**) Flow cytometry for huCD45+ cells in blood of mice after 1 month of injection with CD3-depleted restored cells. (**E**) qPCR for huGAPDH at 2 months after injection with CD3-depleted restored cells, mean ± SD (*n* = 5); ND = not detected. (**F**) CFU-GM and BFU-E in BM at 1 month post-restoration, mean ± SD, *n* = 5. ^*^p ≤ 0.05 vs. mice with unrestored cells. (**G**) Survival curve spanning the study period. (**H**) At yr 1, qPCR for GFP with cells from femurs. The results presented as fold change in which the lowest value was assigned 1, mean ± SD, 4/group. (**I**) CSCs, co-cultured with restored MPBs (− or + NK cells) for 24 h. % CSCs (GFP+) were determined by microscopy and flow cytometry, mean ± SD, *n* = 4. (**J**) Protocol for NSG with dormant CSCs given restored CD3(−) MPB (−/+ NK cells). (**K**–**M**) GFP (surrogate of CSCs) in paraffin section of femurs, −/+ restored MPB: -MPB (**K**), +MPB (**L**), MPB without NK cells (**M**). (**N**) Summary: Aged hematopoietic cells instructed young cells to produce specific miRNA containing MVs to induce MYC and E2F targets to restore the aged MPBs, leading to increased NK activity. Transplantation of restored cells decreased hallmarks of aging: ↑lymphoid:myeloid, ↓senescence/inflammation. See also Supplementary Figure 6.

Age-related defects in NK cells can compromise surveillance for emerging malignancy, infection and induce cell senescence [36, 52]. We asked if the restoration process corrected the age-linked NK defects. Flow cytometry indicated 8-fold more CD56+ cells within the restored cells, relative to unrestored cells (Figure 6A). This correlated with significant (*p* < 0.05) increase in Lytic Units (Supplementary Figure 6A) in 3/4 aged restored cells (Figure 6B).

We further examined the function of the restored NK cells. We selected a model of dormant breast cancer (BC) cells (BCCs) in nude BALB/c [53] (Figure 6C). At 1 month, the mice with CD3-depleted restored MPBs were chimeric for human hematopoietic function with no evidence of lethargy, no palpable lymph node and comparable survival between groups (Figure 6D–6G). Since the BCCs were transfected with pOct4a-GFP, we evaluated the mice femurs for GFP by qPCR. The results indicated 6 folds more GFP+ cells in mice given unrestored MPBs, related to mice with restored aged MPBs (Figure 6H). We did not detect GFP+ cells in the blood, indicating that the dormant cells remained in the femur (Supplementary Figure 6B and 6C). Together, these findings showed the restored MPBs capable of clearing dormant BCCs in the BM.

Since the dormant BCCs were cleared in nude mice, we performed cause-effect studies to determine if restoration of NK functions could explain the dramatic decrease in BCCs. First, we performed *in vitro* studies by co-culturing BC stem cells (CSCs/GFP+) with restored aged MPBs, with or without NK cells (Supplementary Figure 6D). The number of GFP+/CSCs was significantly (*p* < 0.05) reduced with NK-containing restored MPBs as compared to NK-depletion (Figure 6I). Adapting these studies in NSG mice (Figure 6J), we could not detect GFP+ cells in mice given restored NK+ cells whereas control mice (baseline) and mice given NK- restored cells showed dormant CSCs (green) in femurs (Figure 6K–6N). In summary, NK cells in restored MPBs effectively targeted and mostly cleared dormant BCCs *in vivo*.

## DISCUSSION

We report on functional improvement of the aged hematopoietic system, which occurred when the aged cells interacted with the secretome of young hematopoietic cells (UCB and MPBs). The restoration process improved cell senescence, lymphoid:myeloid ratio, inflammation, and NK numbers/function to enhance the innate immune compartment (Figure 6). The *in vitro* findings were successfully recapitulated in NSG mice carrying an aged human hematopoietic system. This was accomplished when we transplanted *in vitro* restored autologous aged MPBs or introducing aged MPBs transfected with restorative miRNAs. Thus, these findings open future translational studies to deliver the restorative miRs.

The evidence indicated long-term hematopoietic competence in mice as evidenced by the ability of CD34+ hematopoietic cells from the restored mice to reconstitute the hematopoietic system of naïve NSG (Figure 3A and 3B). Applying multiple methods, we concluded independence between the restorative process and MHC-II variation between donors (Supplementary Figure 1). More importantly, the released MVs did not express MHC-II (Supplementary Figure 1), indicating the future safety of MVs within the heterochronic cultures for clinical application such as hematopoietic and other tissue regeneration.

We noted increased cell survival of the aged MPBs when they are placed in the heterochronic cultures, which is consistent with enhanced function. It appears that cell restoration might be partly explained by partial reprograming, based on increased MYC pathways and decreased p53 [29]. Partial cellular reprogramming would imply dedifferentiation of hematopoietic cells into cells with stemness. We however argue against partial reprogramming since there was no evidence of malignancy in the mice subjected to long-term observation. A possible mechanism for the improved lymphoid:myeloid ratio in mice is based on other studies showing increased myeloid-associated genes in aged lymphoid cells [54]. Thus, it is possible that during restoration, the increased myeloid genes in lymphoid progenitors could revert to functionally young lymphoid cells.

Lymphoid CD19+ B-cells were increased in femurs and blood of mice when they were transplanted with restored cells, miR cultured or heterochronic cultures. Interestingly, there was less CD19+ cells in mice’s spleen, indicating relatively reduced B-cells (Supplementary Figure 3). This was not in line with evidence of high activity in the prominent follicles of spleen. This led us to propose rapid exit of B-cells from the spleen during *in vivo* restoration to the peripheral circulation.

The miRNA cargo in MVs from heterochronic cultures were responsible for restoring the aged hematopoietic system. The aged cells are likely dictating the miR contents of the MVs within the heterochronic cultures. Prior to being placed in the heterochronic culture, the young (MPBs and UCB) cells exhibited distinct miRNA profiles (Figure 3). After exposure to aged MPBs, both types of young cells express similar miR profiles but distinct from their baseline contents. Thus, the results indicated that baseline young MVs would not be adequate to restore the hematopoietic system and such efficiency requires MVs from heterochronic cultures. Our ongoing studies indicated that we could accomplish *in vivo* hematopoietic restoration with MVs, purified from heterochronic cultures. Based on these studies, we deduced that the *in vivo* restoration was not the result of the injected *in vitro* restored cells but rather, their secretome. Indeed, we showed continued release of the restorative miRNA in the restored age MPBs (Supplementary Figure 4O). These findings warrant further studies to evaluate a treatment with the restorative miRNA directly into aged cells. Also, we were deliberate in transfecting with premiR because of the short life within the cells. The degradation of the premiR would indicate that the miRNA begins a chain of molecular interaction within the cells leading to restoration of the aged cells. These are exciting findings for us to examine other pathways for future treatment.

Age is generally associated with increases in the senescence program. Since plasma from huNSG showed a strong correlation between hematopoietic restoration and improved senescence, this suggested that *in vivo* restoration might have extended to the BM microenvironment and its non-hematopoietic components (Figures 2 and 5). The increase in NK numbers with restoration might have several functional benefits such as the elimination of senescence cells [36]. Thus, restoring HSC competence would lead to continued differentiation of functional NK cells to rapidly eliminate emerging senescence cells as they emerge in various organs. Going forward, we plan to combine a bar-coding system with single cell sequencing to understand restoration in real time and to track the various cell lineages. The data from protein and cDNA senescence arrays were confirmed by RNA-Seq, which showed decreases in inflammatory pathways and other aging hallmarks [55] (Figure 2). The noted decrease in PU.1/SP-1 would improve age-mediated inflammation [56].

Despite transplanting restored MPBs within a milieu of an aged human hematopoietic system, we observed hematopoietic competence that slightly surpassed mice transplanted with young CD34+ cells and significantly more than naïve mice given unrestored cells (Figure 2). This led us to surmise that the *in vitro* restored cells could be involved in regenerating the aged BM microenvironmental hematopoietic and non-hematopoietic supporting cells. Restoration-mediated decrease in PAX5 is consistent with reduced myeloid CFU-GM as well as its role in early B-cell and cellular development [34]. Since PAX5 and PPM1F are regulated by the restorative miR (Figures 5 and 6), it is important for future studies to dissect these pathways since this would provide insights into hematopoietic restoration.

Interestingly, the evidence indicated that the restored cells were behaving as the young cells because they were able to restore the aged MPBs (Supplementary Figure 4O). Thus, one can premise that MVs from the transplanted cells would continue to secrete MVs to impart *in vivo* restoration of the aged hematopoietic cells. If this was the sole method of *in* *vivo* restoration, then competent HSC function would not be explained by expansion during the *in vitro* restoration. Rather, the *in vivo* restoration was mediated by the transplanted cells, which showed experimental evidence of cells with young functions, indicated by omics and confirmatory functional studies. The LTC-IC assay, which is a surrogate of HSC function [38], showed sustained competence of the primitive hematopoietic cells during restoration, which contrasted mice transplanted with aged MPBs from isochronic cultures (Figure 1C).

Sequencing of restored cells identified MYC-linked targets as the only decreased pathways in aged cells, which was reversed with restoration (Figure 2B–2D). This was an intriguing observation since this increase correlated with decreased p53 pathways (Figures 1 and 2). Pharmacological inhibitors verified the roles for MYC and p53 in hematopoietic restoration (Figure 2G). Long-term observation of mice showed sustained health with no evidence of hematological malignancy, thereby arguing against full reprogramming as for induced pluripotent stem cells.

Increased E2F targets in the restored aged MPBs pointed to the involvement of cell cycle in the restoration process, particularly since G1 phase of the cell cycle occurs with silenced E2F activity (Figure 2) [57]. Indeed, our results indicated increases in cycling genes linked to E2F and MYC (Figure 2). MYC could also control E2F switch as a method to regulate the cell cycle of hematopoietic cells during restoration [57]. These findings are intriguing and support the basis for future studies to track the behavior of cells as they transition to restoration. RNA-Seq analyses indicated an increase in G2M pathway, which would facilitate the cells to undergo DNA repair during restoration. Although p53 was decreased during restoration, DNA repair was not compromised since there were increases in other repair genes (Figure 1J). Increased p53 in the aged cells is consistent with enhanced apoptosis identified in the omics study (Figure 2).

Similar CD34+/CD38- progenitors between young and aged MPBs could be explained by the highly proliferative state of aged HSCs [8, 58]. The transplanted restored cells may have changed the BM niche to provide HSCs with a functional microenvironment thereby reducing the age-linked secretome, similar to occurrences in the aged intestinal system [59, 60].

Aging is associated with reduced clonal hematopoietic cell heterogeneity, providing an advantage to the emerging clones with mutated genes [12]. We have not determined if the restoration increased hematopoietic clonal diversity. We can extrapolate such benefit because of increased NK cells and HSC competence for long-term health benefit to the immune/hematopoietic system. An interesting finding is an apparent role for PAX in aging. The aging population has decreased lymphoid cells, yet PAX was linked to the aged function. This suggested that PAX could be a negative regulator of lymphopoiesis, which might be negated by the restorative miRNA. Going forward, it is important to identify the targets of PAX using techniques such as global chromatin immune precipitation assays. As we identified the specific cell types needed to restore the aged hematopoietic system, our planned RNA-Seq will be able to determine what cell type express PAX.

The *in vitro* studies were efficient in determining a key role for MVs in the restoration processes. We also showed continued release of the restorative miRs in MVs from the restored aged cells. Hematopoietic restoration in NSG mice carrying an aged hematopoietic system was achieved when a second transplant occurred with miR-transfected aged MPBs. While MVs, along with the other transplanted hematopoietic cells, are expected to mediate the *in vivo* restoration, the mechanism could occur by MVs activating endogenous BM cells as well as the development of a network comprise of soluble and insoluble factors. The *in vivo* restoration was long-term, suggesting that the restoration may also involve the hematopoietic niche such as stromal cells. We are in the process of depleting different cell subsets in young MPBs to identify those involved in the restoration. Our pilot studies indicated that CD3 depletion enhanced the restoration and showed a need for accessory cells since CD34+ cells alone could not restore the aged cells. We are in the process of expanding on our depletion analyses and once we identified the causative cells, we will then begin to deplete subsets in the aged MPBs. After this, we will repeat the omics studies with the narrowed cell subsets and then knockdown ≥1 of the identified miRs to determine if they are responsible for cell-mediated restoration.

Figure 6N shows a summary of the main findings - MVs with specific miR cargo induce hematopoietic restoration, partly through increases in E2F/MYC and decreased p53 pathways. The restored hematopoietic system reverses its aging hallmarks, including increased NK activity. The summary figure shows the translational potential to the described findings, including the potential for enhancement of the immune system that is needed for vaccine response. The latter is particularly significant considering the current pandemic to facilitate responses of the aged to vaccine. The benefit to medicine is not limited to vaccine response but to improve the surveillance system to eliminate emerging malignancies, enhanced neural health and rapid response to infection. Perhaps it would be prudent to determine how the present technology might be important to enhance the current immune therapies.

## MATERIALS AND METHODS

**Table:** Key Resources Table.

**Reagent or Resource**	**Source**	**Identifier**
**Antibodies**		
Anti-Human PTEN (WB)	Cell Signaling	Cat #9559
Anti-Human AKT (WB)	Cell Signaling	Cat #9272
Anti-Human phospho-GSK3β (WB)	Cell Signaling	Cat #9336
Anti-Human CDK4 (WB)	Cell Signaling	Cat #12790
Anti-Human Cyclin E (WB)	Cell Signaling	Cat #4129
Anti-Human Cyclin D1 (WB)	Cell Signaling	Cat #2978
Anti-Human Cyclin A (WB)	Santa Cruz	Cat #sc-271682
Anti-Rabbit IgG-Rhodamine (FACS)	Santa Cruz	Cat #sc-2367
Anti-Mouse IgG-PE (FACS)	Santa Cruz	Cat #sc-3739
Anti-Human p53 (WB)	Abcam	Cat #ab131442
Anti-Human Ki67 (IHC)	Abcam	Cat #ab15580
Anti-Human Vinculin (WB)	Abcam	Cat #ab91459
Anti-Human Fibronectin (WB)	Millipore-Sigma	Cat #F3648
Anti-Human Pan Cytokeratin (IHC)	Millipore-Sigma	Cat #C2562
Anti-Human β-actin (WB)	Millipore-Sigma	Cat #A5441
Anti-Rabbit IgG-HRP (WB)	Thermo Fisher Scientific	Cat #A16110
Anti-Mouse IgG-AlexaFluor 405 (IHC)	Thermo Fisher Scientific	Cat #A-31553
Anti-Mouse IgG (FACS)	Thermo Fisher Scientific	Cat #31160
PerCP-Cy5.5 Anti-Human CD45 (FACS)	BD Biosciences	Cat #564105
FITC Anti-Human CD45 (FACS)	BD Biosciences	Cat #555482
APC Anti-Human CD45 (FACS)	BD Biosciences	Cat #555485
APC Anti-Human CD34 (FACS)	BD Biosciences	Cat #555824
FITC Anti-Human CD38 (FACS)	BD Biosciences	Cat #555459
APC Anti-Human CD3 (FACS)	BD Biosciences	Cat #555335
PerCP-Cyanine5.5 Anti-Human CD3 (FACS)	BD Biosciences	Cat #561557
PE Anti-Human FLA-DR (FACS)	BD Biosciences	Cat #555812
PE Anti-Human CD56 (FACS)	BD Biosciences	Cat #555516
PE Anti-Human CD4 (FACS)	BD Biosciences	Cat #555347
APC Anti-Human CD33 (FACS)	BD Biosciences	Cat #561817
APC Anti-Human CD8 (FACS)	BD Biosciences	Cat #555369
PE Anti-Human CD19 (FACS)	BD Biosciences	Cat #555413
PE Anti-Rabbit IgG (FACS)	BD Biosciences	Cat #558553
Goat Anti-Rabbit IgG-AlexaFluor 405 (IHC)	ThermoFisher Scientific	Cat #A-31556
Goat Anti-Rabbit IgG Rhodamine (IHC)	ThermoFisher Scientific	Cat #31670
DAPI (IHC)	ThermoFisher Scientific	Cat #D1306
MitoSox™ (IHC)	ThermoFisher Scientific	Cat #M36008
**Biological Samples**		
Human chronic myeloid leukemia bone morrow K562 cell	ATCC	Cat #CCL-243
Human metastatic breast adenocarcinoma MDA-MB-231 cell	ATCC	Cat #HTB-26
**Chemicals, Peptides, and Recombinant Peptides**		
RPMI-1640	Millipore Sigma	Cat #R0883
DMEM with high glucose	Millipore Sigma	Cat #D5796
L-Glutamine	Millipore Sigma	Cat #G8540
Penicillin-Streptomycin	Millipore-Sigma	Cat #P0781
β-mercaptoethanol	Millipore-Sigma	Cat #444203
Hank’s Balanced Salt Solution with Calcium and Magnesium	Millipore-Sigma	Cat #55037C
Fetal Calf Serum	Millipore-Sigma	Cat #F2442
DNase I	Millipore-Sigma	Cat #69182
Ficoll Hypaque	Millipore-Sigma	Cat #H8889
HLM006474	Millipore Sigma	Cat #324461
BCI-137	Millipore Sigma	Cat #531552
Dimethyl Sulfoxide	Fisher Scientific	Cat #BP231-100
Sodium Chloride	Millipore Sigma	Cat #S9888
Magnesium Chloride	Millipore Sigma	Cat #63036
Glycerol	Millipore Sigma	Cat #356352
NP-40	Millipore Sigma	Cat #492016
Phosphate-Buffered Saline	Millipore Sigma	Cat #D1408
Tween 20	Millipore Sigma	Cat #9005-64-5
Triton-X 100	Millipore Sigma	Cat #T8787
Bovine Serum Albumin	Millipore Sigma	Cat #A3912
Protease Inhibitor	Millipore Sigma	Cat #5056489001
Phosphatase Inhibitor	Millipore Sigma	Cat #P5726
CarboxyFluorescein Succinimidyl Ester	ThermoFisher Scientific	Cat #46410
Sybr Green PCR Master Mix II	ThermoFisher Scientific	Cat #4309155
Tris-HCl	Fisher Scientific	Cat #BP153
Exosome-depleted FBS Media Supplement	System Biosciences	Cat #EXO-FBS-250A-1
Recombinant Human GM-CSF	R&D Systems	Cat #215-GM
IL-3	R&D Systems	Cat #203-IL
Erythropoietin	R&D Systems	Cat #287-TC
JQ1	Medchemexpress	Cat #HY-13030
CellTracker Blue CMAC Dye	ThermoFisher Scientific	Cat #C2110
Idasanutlin	MedKoo Biosciences	Cat #205917
**Critical Commercial Assays**		
Total Exosome Isolation Reagent	ThermoFisher Scientific	Cat #4478359
Exosome-Human CD63 Isolation/Detection Reagent	ThermoFisher Scientific	Cat #10606D
SuperSignal™ West Femto Maximum Sensitivity Substrate	ThermoFisher Scientific	Cat #34094
CD34 microbead, human kit	Miltenyi Biotec	Cat #130-046-703
CD56 microbead, human kit	Miltenyi Biotec	Cat # 130-050-401
Mouse Cell Depletion Kit	Miltenyi Biotec	Cat #130-090-858
RNeasy Mini Kit	Qiagen	Cat #74104
Human Aging RT² Profiler PCR Array	Qiagen	Cat # 330231
HiPerfect Reagent	Qiagen	Cat # 301705
miRCURY RNA Isolation Kit	Exiqon	Cat # 300112
NEBNext^®^ Poly(A) mRNA Magnetic Isolation Module	New England BioLabs	Cat # E7490L
Amaxa P3 Primary Cell 4D-Nucleofector X Kit	Lonza	Cat # V4XP-3024
7-AAD/CFSE Cell-Mediated Cytotoxicity Assay Kit	Cayman Chemical	Cat # 600120
CellTiter-Blue^®^ Cell Viability Assay	Promega	Cat #G8080
**Experimental Models: Organisms/Strains**		
NSG Mice	The Jackson Laboratory	Cat #5557
BALBc Mice	The Jackson Laboratory	Cat #000651
**Oligonucleotides**		
Primer: GAPDH, Human Forward: CAGAAGACTGTGGATGGCC	Life Technologies	N/A
Primer: GAPDH, Human Reverse: CCACCTTCTTGATGTCATC	Life Technologies	N/A
Primer: GAPDH, Human and Mouse Forward: AGTCCCCCACCACACCACTGAAT	Life Technologies	N/A
Primer: GAPDH, Human and Mouse Reverse: TTGATGGTACATGACAAGGTGC	Life Technologies	N/A
Primer: CASP14 Forward: GTTCCGAAGAAGACCTGGAT	This Paper	N/A
Primer: CASP14 Reverse: TTCTCCAGCTTGACCATCTC	This Paper	N/A
Primer: GALNT6 Forward: GGAGCACCTAAAGGAGAAGC	This Paper	N/A
Primer: GALNT6 Reverse: CTGTCTTGTCCTCAGCGATT	This Paper	N/A
Primer: PAX5 Forward: CATCCGGACAAAAGTACAGC	This Paper	N/A
Primer: PAX5 Reverse: ACCGGAGACTCCTGAATACC	This Paper	N/A
Primer: PPM1F Forward: CTTGTCTGACCCTGTGAACC	This Paper	N/A
Primer: PPM1F Reverse: CTTGGCTTTCCTGAGAAACA	This Paper	N/A
Primer: SUMO2 Forward: ATGGTTCTGTGGTGCAGTTT	This Paper	N/A
Primer: SUMO2 Reverse: CTGCTGTTGGAACACATCAA	This Paper	N/A
Primer: β-actin Forward: ATCCTCACCCTGAAGTACCC	This Paper	N/A
Primer: β-actin Reverse: AGCCTGGATAGCAACGTACA	This Paper	N/A
**Software and Algorithms**		
RNA-protein Association and Interaction Networks v1.0	Junge et al., 2017	https://rth.dk/resources/rain/
Partek^®^ Genomics Suite^®^ software v7.0	Partek Inc., 2018	https://www.partek.com/
STRING, v11.0	Szklarczyk D et al., 2019	https://string-db.org/
CLC Genomics Workbench, v11.0	Qiagen	N/A
TargetScan v7.0	Agarwal et al., 2015	http://www.targetscan.org
TarBase v8.0	Karagkouni et al., 2018	http://diana.imis.athena-innovation.gr
BD CellQuest Pro™	BD Biosciences	N/A
UN-SCAN-IT Gel™	Silk Scientific, Inc.	https://www.silkscientific.com/index.htm
FlowJo	BD Biosciences	https://www.flowjo.com/solutions/flowjoRRID:SCR_008520
Adobe Photoshop	Adobe	https://www.adobe.com/products/photoshop.htmlRRID:SCR_014199
BCL2FASTQ	Illumina	https://support.illumina.com/sequencing/sequencing_software/bcl2fastq-conversion-software.htmlRRID:SCR_015058
RT² Profiler PCR Array Human Senescence	NCBI Genome Expression Omnibus	GSE141838
RT² Profiler PCR Array Human Aging	NCBI Genome Expression Omnibus	GSE141837
RNA sequencing	NCBI Genome Expression Omnibus	GSE138563
miRNA sequencing	NCBI Genome Expression Omnibus	GSE138564
**Other**		
Evos Fl Auto 2	Thermo Fisher Scientific	AMAFD2000
7300 Real Time PCR System	Thermo Fisher Scientific	7300
Qubit Instrument	Thermo Fisher Scientific	Q33226
Tapestation 2200 Instrument	Agilent	G2965A
D1000 Screentapes	Agilent	5067–5582
Illumina’s Nextseq 500	Illumina	Serial # NB500952
Synergy HTX-Multi-Mode Reader	Biotek	Synergy HTX
FACS Calibur	BD Biosciences	E4400

### Cell lines

K562 and MDA-MB-231 were cultured as per American Type Culture Collection instruction. The cell lines were tested by Genetica DNA Laboratories (Burlington, NC) and were deemed to be the original cells using ATCC STR database (https://www.atcc.org/search-str-database).

### Human subjects

The use of de-identified human mobilized peripheral blood (MPB), peripheral blood (PB) and umbilical cord blood (UCB) was approved by Rutgers Institutional Review Board (IRB), Newark, NJ, USA.

#### MPB

MPB was collected from aged (>60 yrs) and young (<30 yrs) donors (Supplementary Table 1). Aliquots (10–20 mL) of MPB for A5–A8 (aged) and Y6-Y8 (young) were donated for research purposes through Progenitor Cell Therapy (Allendale, NJ, USA). Donors A1-A4 (aged) and Y1-Y5 (young) were recruited, mobilized and subjected to leukapheresis by HemaCare Corporation (Van Nuys, CA, USA). HemaCare was approved as a collection site by Rutgers IRB. The site is FDA-registered, AABB-accredited and operated under GMP-compliance. Study participants were given subcutaneous Neupogen^®^ (G-CSF) at 5 μg/kg/day for 5 days. On day 6, MPB was collected using the Spectra Optia^®^ Apheresis System to process 18 L of blood at a flow rate of 50 to 100 mL per min. Immediately after mobilization, the MPB were shipped to Rutgers.

#### Umbilical cord blood (UCB)

The demographics obtained for 10 UCB samples are shown in Supplementary Table 1. The samples were provided within 24 h of collection by the Community Blood Services (Montvale, NJ, USA), which is an AABB-accredited blood bank registered with the FDA. The mononuclear fraction was isolated by Ficoll-Hypaque density gradient and then cryopreserved for later use.

#### Peripheral blood (PB)

PB was obtained from healthy donors (18–30 yrs) and the mononuclear fraction isolated by Ficoll Hypaque density gradient.

### Isolation of CD34^+^ cells and cell cryopreservation

Total MPB, herein referenced as total nucleated cells (TNC), were cryopreserved by adding chilled cryopreservation media to the cell suspension at a 1:1 ratio while gently shaking the cells. The resuspended TNC was ~ 5 × 10^7^ cells/mL. Cells were stored using a controlled rate freezer (Cryo Met Freezer, Thermo Fisher) at −1°C/min until the temperature reached -100°C. After this, the cells were transferred into liquid nitrogen.

CD34^+^ cells were selected using the CD34 microbead human kit (Miltenyi, Auburn, CA, USA). The method followed manufacturer’s instructions. Briefly, TNC was pelleted at 4°C, 10 min, 500 *g*. The TNCs were resuspended in cold MACS buffer at a concentration of 10^8^ cells/300 μL buffer. Cell suspension was incubated in 100 μL of FcR Blocking Reagent and CD34 MicroBeads for 30 min at 4°C. After this, cells were washed with MACS buffer and the CD34^+^ cells were positively selected by magnetic separation with LS columns. The purified CD34^+^ cells were immediately cryopreserved as described above.

### Transwell assay

Non-contact cultures were performed in 6- or 12-well transwell system, separated by a 0.4 μ membrane (BD Falcon, ThermoFisher). In the case of 6-well plates, the inner wells contained 10^7^ young MPB or CB with the same number of aged MPBs in the outer wells. The numbers were scaled lower for 12-well plates. At optimization, cells were cultured for up to 5 wks in RPMI 1640 supplemented with 10% FCS, 2 mM glutamine and 0.5 μM β-mercaptoethanol (β-ME) (R10 media). Cultures with allogeneic cells were designated heterochronic and those with autologous cells, isochronic. During the culture period, 50% percent of cell-free media were replaced every four days with fresh R10 media without cell passaging. Except for FCS, we did not supplement the media until we were ready to evaluate aged hematopoietic activity in clonogenic assay. The latter contained supplement (see below for method). In this case, we did not mask the restoration with exogenous growth factors. Based on the optimization data, all other transwell cultures were conducted for 1 wk.

### Cell migration from inner to outer wells

Transfer of cells from the inner to the outer wells of co-cultures were studied as follows: young MPBs were labeled with CarboxyFluorescein succinimidyl ester (CFSE) (Thermo Fisher) following manufacturer’s recommended protocol. Briefly, CFSE was diluted in 1x PBS to 5 μM (stock solution) and then diluted at 1/1000 (working solution). The latter was used to label cells by incubating for 20 min at room temperature. The labeled cells were added to the inner wells of the heterochronic cultures and at weekly intervals, the cells in the outer wells were analyzed for CFSE by flow cytometry.

### Flow cytometry

Cells (10^6^) were labeled with the primary antibodies in 100 μl PBS. Each labeling used an isotype IgGs for background labeling (control) at the same concentration as the primary antibodies (see above for concentrations). After 30 mins at room temperature, the cells were washed with PBS and acquired on a FACSCalibur flow cytometer (BD Biosciences). Data were analyzed using BD CellQuest Pro™ software (BD Biosciences).

### Western blot analyses

Cell extracts were isolated with cell lysis buffer (50 mM Tris-HCl (pH 7.4), 100 mM NaCl, 2 mM MgCl_2_, 10% glycerol, and 1% NP-40) as described (Ghazaryan et al., 2014). Extracts (15 μg protein) were electrophoresed on SDS-PAGE and then transferred to polyvinylidene difluoride membranes (Perkin Elmer). The membranes were incubated overnight at 4°C on rocker with primary antibodies in 5% milk dissolved in 1x PBS tween. Next day, the membranes were incubated with species-specific HRP tagged secondary IgG in the same diluent. After 2 h, the membranes were developed by chemiluminescence using SuperSignal West Femto Maximum Sensitivity Substrate (Thermo Fisher Scientific).

### Clonogenic assays

Clonogenic assays for hematopoietic progenitors, granulocytic-monocytic (CFU-GM) and early erythroid (BFU-E), were performed as described in methylcellulose matrices (Rameshwar et al., 2001). Cultures for CFU-GM contained 3 U/mL GM-CSF and BFU-E, 3 U/mL rhIL-3 and 2 U/mL Epo. Colonies were counted by a blinded observer. Each colony contained >15 cells.

### Modified long-term culture-initiating cell (LTC-IC) assay

Confluent stromal cells in the outer well of the transwell cultures were subjected to 1.5 Gy, delivered by a cesium source. After 16 h, the floating cells were washed and 10^7^ BM mononuclear cells added to the γ-irradiated stroma. At wks 6, 10 and 12, aliquots of mononuclear cells were assayed for CFU-GM in clonogenic assays (see above) [61].

### Mixed lymphocyte reaction (MLR)

One-way mixed lymphocyte reaction (MLR) was performed as described previously [62]. Briefly, γ-irradiated (20 Gy) stimulator cells from isochronic or heterochronic cultures were added to 96 well-flat bottom plates (Corning, Corning, NY) at 10^6^/mL in 0.1 mL volume in triplicates. Autologous responder freshly thawed cells were added at the same concentration and volume. Control MLR used peripheral blood mononuclear cells (PBMCs) from two different donors (20–30 yrs). The MLR reactions were incubated at 37^o^C. At 72 h, the wells were pulsed with 1 μCi/well of [methyl-^3^H]TdR (70–90 Ci/mmol; NEN Radiochemicals, PerkinElmer, Akron, OH). After 16 h, the cells were harvested with a PhD cell harvester (Cambridge Technologies, Bedford, MA) onto glass-fiber filters, and [^3^H]TdR incorporation quantified in a scintillation counter (Beckman Coulter, Brea, CA, USA). The results are expressed as the stimulation index (S.I.), which is the mean dpm of experimental cultures (responders + gamma-irradiated stimulators)/dpm of responder cells cultured in media alone.

### Cell titer blue viability assay

The cells in the outer wells of transwell cultures were assayed for viability with CellTiter-Blue Cell Viability Assay kit (Promega, Madison, WI). Viability analyses followed manufacturer's instructions. Briefly, CellTiter-Blue reagent was added to aliquots of cells in 96-well plates. The plates were incubated for 4 h at 37°C and then read on a fluorescence microplate reader at 560 nm excitation/590 nm emission. Percent viability was calculated from a reference using untreated healthy cells, which were considered 100% viable, and cell-free wells containing reagent alone, which were considered 0% viable.

### Real time PCR for human cells in mouse femur

RNA extraction was performed according to manufacturer’s protocols with the RNeasy Mini Kit (Qiagen, Germantown, MD). Quality and concentration of RNA were determined with the Nanodrop ND-1000 spectrophotometer (ThermoFisher Scientific). The High-Capacity cDNA Reverse Transcription Kit (Life Technologies) was used to convert RNA to cDNA and 10 ng used in real-time PCR with Sybr Green PCR Master Mix II (Life Technologies) using primers from human and mouse GAPDH (see above).

### PCR array

Total RNA (2 μg) was extracted from MPB with the RNeasy Mini Kit (Qiagen) and then reverse-transcribed using the First Strand cDNA Synthesis Kit (Life Technologies). The cDNA (200 ng) was used for quantitative PCR with the Human Cellular Senescence RT² Profiler™ PCR Array (Qiagen) containing 84 key genes involved in the initiation and progression of the biological process causing cells to lose the ability to divide. Prepared cDNA templates were added to the ready-to-use PCR master mix at equal volumes within each well of the same plate. The arrays were then run on the 7500 Real Time PCR System (Life Technologies) with the cycling profile (40 cycles) as follows: 94°C for 15 secs and 60°C for 45 secs. The data were analyzed with Qiagen PCR Array Data Analysis Software and normalized to five housekeeping genes provided within each array. Controls were also included for genomic DNA contamination, RNA quality, and general PCR performance. The normalized data are presented as fold difference, with a value of 1 representing no change. Differential and overlapping miRNAs are presented in Venn diagram. The overlapping areas include miRNAs with <1.5-fold difference among groups.

### MV isolation and nanoparticle tracking analysis

MVs were isolated from cell culture media as described (Bliss et al., 2016). In addition, MVs were also isolated with the Total Exosome Isolation Kit (Life Technologies), using a modified version of the manufacturer’s protocol. Specifically, isochronic and heterochronic cultures were established with Exosome-depleted FBS Media Supplement (System Biosciences, Palo Alto, CA, USA). Culture media collected on the 4th and 7th days were pelleted and supernatant transferred to another tube for further clarification at 2000 *g* for 30 min to remove residual cells and debris. The remaining supernatant was transferred to a fresh tube and 0.5 volumes of Total MV Isolation reagent was added for overnight incubation at 4°C. The following day, samples were centrifuged at 10,000 *g* for 1 h at 4°C to pellet the exosomes for subsequent nanoparticle tracking analysis (NTA) or long-term storage at −80°C. Analysis of absolute size distribution of MVs was performed using the NanoSight LM10 with NTA3.1 software (Malvern Panalytical, Malvern, UK). Particles were automatically tracked and sized based on Brownian motion and the diffusion coefficient. For NTA, MVs were re-suspended in 0.5 mL of PBS and measured using the following parameters: Temperature = 25.6 +/− 0.5°C; Viscosity = (Water) 0.99 +/− 0.01 cP; Measurement time = 30 sec; Syringe pump speed = 30. The detection threshold was similar in all samples. Three recordings were performed for each sample.

### MV entry into aged cells

Purified MVs (4 × 10^6^/500 μL) from 3-day co-cultures were resuspended in PBS containing 25 μM CellTracker Blue CMAC Dye (ThermoFisher Scientific). The MVs were incubated for 45 min at 37°C. After this, MVs were washed as follows: Increased PBS to 5 mL followed by ultracentrifugation at 130,000 xg for 80 mins in the cold. The MVs were then added to naïve aged MPBs and then subject to timeline (24 and 48 h) for cellular uptake. The latter was assessed by the following methods: Confocal 3D imaging using Olympus Fluoview FV10i and the images processed with the FV10-ASW software, Version 04.02.03.06; 2D imaging with the EVOS FL Auto2 (Invitrogen/ThermoFisher Scientific).

### MitoSox assay

MPB cells (10^6^) were labeled with anti-CD34-APC and -CD45-PerCp-Cy5.5, as described below for flow cytometry. The cells were washed and incubated with 5 μM MitoSox™ Red for 10 min at 37°C in the dark and then washed again with warm HBSS. The data was acquired and analyzed with the FACSCalibur.

### Senescence Associated Secretory Phenotype (SASP) array

#### Media

Senescence associated secretory factors (SASFs) in media from heterochronic cultures at day 1 and wk 4 were analyzed with Custom C-Series Human Antibody Arrays with 68 different factors linked to cellular senescence [63]. The antibodies were obtained from Ray Biotech (Norcross, GA, USA). Briefly, cell culture media were pelleted at low speed centrifugation (300 *g*) to remove cell debris and stored in siliconized microfuge tubes at −80°C until assayed. Incubation and detection of factors within the conditioned media followed the manufacturer’s suggested protocol. Background levels were calculated by incubating the arrays with complete media alone and then subtracting the obtained values from each conditioned media experiment. Densitometry was performed using the UN-SCAN-IT densitometry software (Silk Scientific; Orem, UT).

#### Plasma

Detection of SASFs in plasma of huNSG mice was performed as described above for media. Briefly, blood from huNSG mice was pelleted for 10 min at 300 *g* and the plasma supernatant collected in siliconized microfuge tubes for SASF determination. Background was subtracted from the density of parallel analyses with plasma from non-humanized NSG mice.

### Senescence and aging gene arrays

Cells were flushed from the femurs of huNSG using a 26-gauge needle. Murine cells were eliminated with a Mouse Cell Depletion Kit. Total RNA (2 μg) was extracted from purified cells using the RNeasy Mini Kit and reverse-transcribed with the RT^2^ First Strand Kit. 20 ng of cDNA was used for qPCR with the Human Cellular Senescence and Human Aging RT² Profiler™ PCR Arrays (Qiagen). Arrays were run on the 7300 Real Time PCR System (Life Technologies) with the cycling profile (40 cycles): 94°C for 15 secs and 60°C for 45 secs. Gene expression analysis was performed using Qiagen PCR Array Data Analysis Software. The data were normalized to five housekeeping genes provided within each array. The data was submitted to the NCBI Genome Expression Omnibus (GEO; https://www.ncbi.nlm.nih.gov/geo/) under SuperSeries accession number GSE138565. Hierarchical clustering and heat map generation were performed with Heatmapper software (http://www.heatmapper.ca/), as described [64].

### MiRNA microarray and qPCR

Total RNA (500 ng) was isolated from MVs using the miRCURY RNA Isolation Kit (Exiqon). RNA was reverse-transcribed with the miScript II RT Kit (Qiagen) and 20 ng of cDNA used for qPCR with the human miFinder miRNA Array (Qiagen). The PCR was done with the following cycling conditions: 94°C for 15 mins, 40 cycles at 94°C for 10 secs, 55°C for 30 secs, 70°C for 30 secs, followed by melt curve analysis. The data were analyzed with the online miScript miRNA PCR Array data analysis tool (Qiagen).

Validation of miRNAs identified in the microarray and NGS was done by individual qPCR experiments using miScript primer assays and similar cycling and analysis schemes. Total RNA (2 μg) was also isolated from cells, as described above, for profiling of downstream miRNA targets by qPCR using the primer pairs listed above with the following cycling conditions: 95°C for 15 mins, 40 cycles at 94°C for 15 secs, 51°C for 30 secs, 72°C for 30 secs, followed by melt curve analysis. Analyses were performed with Qiagen PCR Array Data Analysis Software, as described above. Array and individual qPCR studies were normalized to RNU6, SNORD68 and SNORD95 and presented as fold change. The 30 genes with marked changes following restoration were selected for links to other genes using RNA-Protein Association and Interaction Networks (RAIN) [65].

### RNA-Seq

RNA Seq was performed at the Genomics Center at Rutgers New Jersey Medical School (Newark, NJ, USA). Total RNA was submitted to the center where poly A RNA was purified using NEBNext^®^ Poly(A) mRNA Magnetic Isolation Module (New England BioLabs, Ipswich, MA, USA). Next generation sequencing (NGS) libraries were prepared using the NEB Ultra II Library Preparation Kit and NEBNext^®^ Multiplex Oligos for Illumina (Dual Index Primers Set 1) (New England BioLabs). The generation of the libraries followed manufacturer’s protocol. The libraries were subjected to quality control using Qubit instrument and high sensitivity Kit from Thermo Fisher as well as Tapestation 2200 instrument and D1000 ScreenTapes from Agilent (Santa Clara, CA, USA). The libraries were diluted to 2 nM and then denatured as per Illumina’s protocol and run on Illumina’s NextSeq instrument (San Diego, CA, USA) using 1X75 cycle high throughput kit. The BCL files that were generated from the sequencing were demultiplexed and converted to FastQ files using BCL2FASTQ software from Illumina. All raw and processed sequencing data have been submitted to the NCBI Genome Expression Omnibus (GEO; https://www.ncbi.nlm.nih.gov/geo/) under accession number GSE138563, SuperSeries accession number GSE138565.

### MiRNA sequencing (miR Seq)

Total RNA from MVs and cells was isolated using the miRCURY RNA Isolation Kit with small and large RNAs fractionated with the RNeasy MinElute Cleanup Kit, both according to manufacturer’s recommended specifications (Qiagen). Half of the small RNA fraction (200 ng) was used in library preparation with the NEBNext Multiplex Small RNA Sample Prep Set for Illumina - Set 1 (New England Biotechnology), according to the following protocol: (1) ligation of the 3′ SR Adaptor, (2) hybridization of the reverse transcription primer, (3) ligation of the 5′ SR Adaptor, (4) reverse transcription for first strand cDNA synthesis and (5) PCR enrichment. After PCR, samples were cleaned up and size selection performed. Briefly, 2 μl of sample was subjected to TapeStation (Agilent) analysis to ascertain band sizes. Samples were run on 8% acrylamide gel at 100V for 1 hr, with correct size bands excised for gel purification. Small RNA libraries were diluted to 2 nM and run on a miSeq System (Illumina) for NGS using the V2 kit (Illumina). All raw and processed sequencing data have been submitted to the NCBI Genome Expression Omnibus (GEO; https://www.ncbi.nlm.nih.gov/geo/) under accession number GSE138564, SuperSeries accession number GSE138565.

### Data analyses

#### RNA network

Specific genes from the senescence array data were analyzed for RNA interaction using the RNA-protein Association and Interaction Networks (v1.0) (RAIN) [65]. The upregulated genes following restoration were input into the program. The database projected the output in the form of networks and co-expression graphs.

#### RNA Seq

Partek^®^ Flow^®^ software was used to align the reads to Homo Sapiens assembly hg38. Transcript abundance of the aligned reads were performed with Partek’s (version 7) optimization of the expectation-maximization algorithm (Partek Inc., St. Louis, MO, USA). The data trends and outliers were detected by Principal Component Analysis (PCA). The RNA-Seq data was normalized by using log_2_ counts per million (CPM) with an offset of 1. Batch effect was removed between two sequencing rounds of the same biological sample. Differential analyses were performed using Partek™ GSA with an FDR *q*-value < .05 and a fold change of 1.3 in aged vs. restored, 1.45 in Restored vs. Young, and 1.65 in Aged vs Young. The fold change cutoff was based on point of maximum curvature in the ranked fold changes in each comparison. The analyses resulted in 2,140 genes. The differences in gene expression were visualized by hierarchal clustering using Morpheus. Clusters were then identified by visual analysis as well as dendrogram branches. Top gene ontology (GO) terms for each cluster were found using STRING (Version 11) and the significant pathways and functions were displayed next to the hierarchal clustering [66]. Additionally, pathway and functional analyses were done by Gene Set Enrichment Analysis (GSEA). False discovery rate of ≤0.05 was used as a cutoff to select significantly up or downregulated pathways.

#### Ingenuity pathway analysis (IPA) - hematological effects analysis

Pathways of genes linked to hematological functions were selected using Ingenuity Pathway Analyses (IPA, QIAGEN Inc.,). We ranked the pathways by Significance score, defined as -Log_10_B-H *p* value times activation z-score. We retained the pathways associated with hematological functions that passed a significance score threshold of >2.

#### MiR Seq

Data analyses were performed using CLC Genomics Workbench (Qiagen) according the following data workflow: (a) Fastq files were imported into the analysis suite, (b) sequences were trimmed to remove poor quality and short reads, (c) trimmed reads were run through the Small RNA Analysis pipeline, (d) extraction and counting, (e) annotation and count merging to identify expression level of each mapped miRNA. Mapped reads from individual samples were then compared to determine fold change for each miRNA.

#### MiRNA target prediction and network analyses

All miRNAs exhibiting greater than 100 mappable reads were analyzed. Expression data from NGS was analyzed *in silico * by IPA to predict miRNA targets and downstream signaling networks. Differentially expressed exosomal and intracellular miRNA (1.4-fold cutoff) between young and aged isochronic, and aged isochronic and heterochronic samples were uploaded to the IPA suite for Core Network Analysis. Predicted networks from the Core Analyses were then simultaneously linked using Comparison Analyses to identify the MV-cell interactome during heterochronic restoration. Potential mRNA targets of candidate miRNAs were determined using the miRNA Target Filter. The source of the miRNA-mRNA relationship and the confidence of the relationship predictions were from TargetScan (http://www.targetscan.org/) and the experimentally observed relationships were from TarBase (http://diana.imis.athena-innovation.gr). mRNA target selection was based on target rank score, where the highest ranked targets were common to the most candidate miRNA (score = 6) and the lowest ranked targets to the least candidate miRNA (score = 1). Potential interaction with the MV - cell interactome was evaluated by creating a mock mRNA target expression profile (10-fold downregulation) to generate a Core Analysis network that could be likened using the Comparison Analysis tool. Candidates whose predicted networks converged with the interactome were selected for additional evaluation.

#### Targets of validated miRNAs

Analyses of 6 miRNAs for predicted targets were performed with TargetScan human database. A total of 6101 potential targets were evaluated, with a number of common targets within the group of 6 miRNA displayed within the descending concentric circles. 25 targets were identified that met the following conditions: (1) ≥ 4 common hits among the miRNA group, including miR-619 or miR-1303 or, (2) ≤ 3 common hits among the group, including miR-619 and miR-1303. Predicted expression of these targets was analyzed by IPA and the resulting network predictions compared to the young exosomal and aged heterochronic intracellular miRNA interactome. Selected targets were tabulated and then pared down based on expression in relevant tissues, namely they encode verified protein and, their expression is not limited solely to neural tissue.

#### qPCR miRNA array analyses

Total RNA (500 ng) was isolated from MVs using the miRCURY RNA Isolation Kit and reverse-transcribed with the miScript II RT Kit. 20 ng of cDNA was used for qPCR with the human miFinder miRNA Array, with cycling conditions of 94°C for 15 min, 40 cycles at 94°C for 10 seconds, 55°C for 30 secs, 70°C for 30 secs, followed by melt curve analysis. The data were analyzed with the online miScript miRNA PCR Array data analysis tool (SABiosciences). The miRNAs identified by microarray and NGS were validated by real time PCR with miScript primer assays using similar cycling and analysis schemes. Total RNA (2 μg) was also isolated from cells, as described above, for profiling of downstream miRNA targets by real time PCR using primers listed above and the following cycling conditions: 95°C for 15 mins, 40 cycles at 94°C for 15 secs, 51°C for 30 secs, 72°C for 30 secs, followed by melt curve analysis. Analyses were performed with Qiagen PCR Array Data Analysis Software, as described above.

### Nucleofection of miRNA mimics, miRNA inhibitors and siRNA

Aged cells (10^7^/sample) were nucleofected with miRNA mimics (Qiagen), miRNA inhibitors (Qiagen), negative control siRNA (Qiagen), negative control miRNA inhibitor (Qiagen) or downstream target candidate siRNAs (Origene) using the Amaxa P3 Primary Cell 4D-Nucleofector X Kit (Lonza) on a 4D Nucleofector device (Lonza), according to manufacturer’s specific protocol. Briefly, CD34^+^ cells were nucleofected with 60 nM of total miRNA mimics, miRNA inhibitors or siRNA using the “human CD34^+^ cell” program. Real time PCR for miRNA levels were similar (*p* > 0.05) between restored CD34+ cells and those subjected to nucleofection.

### Sorting of breast cancer stem cells (CSCs)

MDA-MB-231 breast cancer cells were stably transfected with pEGFP1-Oct3/4, as previously described [53]. The CSCs were selected as the top 5% cells with the highest GFP intensity (Oct4^hi^) and then sorted with the FACSAria II cell sorter (BD Biosciences).

### *In vivo* studies

The use of mice was approved by Rutgers Institutional Animal Care and Use Committee (Newark Campus, NJ). Mice were housed in an AAALAC-accredited facility.

#### Humanization (huNSG)

Female NSG mice (NOD.Cg-*Prkdcscid Il2rgtm1Wjl*/SzJNOD-*scid* IL2Rgamma^null^, 5 wks) were obtained from Jackson Laboratories (Bar Harbor, ME). These mice lack the major immune cells such as T-cells, B-cells, macrophages and NK cells. After 1 wk acclimatization, the NSG were humanized as described [67–69]. NSG mice were subjected to 150 cGy whole body γ-irradiation using a Mark-I cesium irradiator unit. At 5 h post-irradiation, the mice were injected intravenously with 5 × 10^5^ human CD34^+^ cells isolated from aged or young MPBs. At 9 and 13 wks post-transplantation, we assessed the mice for chimerism by flow cytometry for human CD45. Blood cells were co-labeled with anti-human CD45-APC and anti-mouse CD45-FITC.

#### In vivo restoration

huNSG with aged CD34+ cells were transplanted by intravenous injection of 5 × 10^5^ autologous aged cells from 7-day CD3-depleted isochronic (non-restored) or heterochronic (restored) cultures. Control mice were injected with sterile PBS. % huCD45+ cells were calculated from total nucleated cells; % huCD34+ cells were calculated from total huCD45+ cells.

#### Serial transplant

Mice were injected with 10^5^ huCD34+ cells taken at the end point of the first set of transplants from the three groups.

#### MiRNA-based in vivo restoration

The miRNA-based studies were performed as above, except for a second injection with 60 nM miR-619, miR-combo (miR-619, -1303, 4497) or control miR. The cells were transfected with HiPerFect reagent and then cultured for 7 days. CD3+-depleted cells were transplanted as above. At the end point of 14−15 wks post-transplant, blood, femurs and spleens were harvested for biochemical, phenotypic and functional analyses. Major organs were also harvested for histological assessment. Tissue embedding, processing and staining were performed by the Digital Imaging and Histology Core of Rutgers-New Jersey Medical School Cancer Center (Newark, NJ). Histologic findings were confirmed on H&E slides by a board-certified veterinary pathologist.

#### NK function in mice with breast cancer stem cell (CSC)

Nude female BALB/c mice (6 wks) were injected with CSCs for established dormancy in femurs as described [53]. The CSCs were isolated as outlined above. Mice with dormant cancer cells were injected with 10^6^ restored or unrestored aged MPBs. After one month, mice were studied for human chimerism and GFP cells were analyzed by real time PCR for GFP. Groups of 10 mice were followed for one year for survival.

The role for NK cell in targeting dormant breast cancer cells in mice was studied with NSG due to the lack of endogenous NK cells. The method followed procedure used for nude BALB/c mice. The only modification is we performed parallel studies by depleting NK cells by phenotype. We validated NK depletion by flow cytometry and by function using cytotoxic assay.

### Immunohistochemistry

Immunohistochemistry for human pan cytokeratin and Ki67 in murine femurs was performed as described [37]. Briefly antigen was retrieved from deparaffinized sections of mouse femurs by heating at 56°C overnight. After this the slides were dewaxed with xylene and ethanol then rehydrated, fixed and permeabilized with 0.1% Triton X. Slides were then washed 3x in 1x PBS and then incubated overnight at 37°C in a humidified chamber with primary antibodies at 1/500 final dilution for anti-cytokeratin and anti-Ki67. The slides were washed and then incubated (2 hrs at room temp) in a humidified chamber with fluorescence-tagged secondary antibodies at 1/1000 final dilution: AlexFluor 405 for cytokeratin and rhodamine for Ki67. The slides were washed then covered with 1x PBS and then immediately analyzed on the EVOS FL Auto 2 Imaging System (ThermoFisher Scientific). Tissues from scraped femurs were placed on slides and then similarly imaged.

### Co-culture between natural killer (NK−/+) and CSCs

NK cell depletion in the restored and unrestored MPBs was conducted with human CD56 microbeads from Millitenyi (Auburn, CA, USA). The method followed manufacturer’s instruction. Co-cultures were performed with 10^5^ breast CSCs and 10^7^ restored MPB, with or without NK cells. After 24 h, the number of CSCs were determined by the number of GFP+ breast cancer cells using fluorescence microscopy (Evos FL2 auto).

### NK cell assay

#### ^51^Cr release

NK cell function was determined by the [^51^Cr] radionuclide assay for cytotoxicity, which was performed as described previously [70]. Briefly, the K562 cell line was used as targets (T), and MPBs as effector (E) cells. K562 cells (5 × 10^6^/ml) were labeled with 200 μCi of ^51^Cr (PerkinElmer, Wellesley, MA). After labeling, the K562 cells were washed three times and then resuspended at 10^5^/mL in RPMI 1640 + 10% FCS. The effector MPBs were resuspended at 10^7^/mL RPMI 1640 + 10% FCS. Equal volumes (100 μl) of effector and target were added to round bottom 96-well Corning plates (Millipore Sigma) in quadruplicates at E:T ratios of 100:1, 50:1, 25:1 and 12.5:1. Spontaneous [^51^Cr] release was determined by incubating targets alone, and total release was determined in parallel incubations with 1% Triton X-100. After 4 h of incubation, cell-free supernatants were collected, and the amount of [^51^Cr] release was determined in a γ-counter. The percentage lysis was calculated as follows: [experimental point (dpm) − spontaneous release (dpm)]/[total release (dpm) − spontaneous release (dpm)] × 100. Values of spontaneous release were <1% of total release. The average technical replicates were plotted on a graph of E:T vs. % Lysis. The lytic units (LU) were determined as described (Bryant et al., 1992). One LU was assigned as the number of effector cells that lysed 20% of the target cells. Total LU is the number in 10^7^ effectors. Each replicate was used to calculate the total LU then plotted on the same experimental point as mean ± SD.

#### Carboxyfluorescein succinimidyl ester (CFSE/7-AAD)

Cytotoxicity assay was performed with CFSE/7-AAD Cell Cytotoxicity kit (Cayman Chemical, Ann Arbor, MI), following manufacturer’s instructions. K562 cells (10^7^) were labeled with CFSE dye for 15 mins and then washed twice. Cells were diluted to 10^5^/ml and incubated for 30 mins at 37°C. Day 7 cultures of restored or unrestored aged MPBs were used as effector cells. The effector and target cells were added into 12-well plates at the following E:T ratios: 0:1, 6.25:1, 12.5:1 and 25:1. The plates were incubated for 4 h at 37°C. Cells were harvested and stained with 7-AAD. Events (50,000) were acquired on FACSCalibur flow cytometer. Data were analyzed using Cellquest pro software (BD Biosciences). Target cells stained only with 7-AAD were used to calculate spontaneous lysis while effector cells stained only with 7-AAD were used to detect dead effector cells. The % lysis was calculated as [Cells positive for both CFSE and 7-AAD/Total CFSE labelled cells] × 100 − spontaneous lysis.

### T-Cell activation assay

T-cell activation was determined using the T-Cell Activation/Expansion Kit. Briefly, anti-biotin MACSiBead Particles were loaded with CD2, CD3, and CD28 antibodies. Cells from 7-day isochronic or heterochronic cultures, or MNCs isolated from huNSG mouse blood by Ficoll-Hypaque density gradient, were incubated with loaded anti-biotin MACSiBead Particles at a 1:2 bead to cell ratio for 72 h to activate T-cells. Addition of unloaded MACSiBead Particles served as negative control. After 72 h, cells were fluorescently labeled using CD45-FITC, CD4-PE, CD25-PerCP-Cy5.5 and CD8-APC to determine T-cell activation status by flow cytometry.

### Statistical analyses

Statistical analyses were performed with ANOVA and Tukey-Kramer multiple comparisons test. For array and NGS expression analyses, average linkage was used for clustering and Pearson correlation analysis used for distance measurement to generate heatmaps and hierarchically cluster genes. *P* < 0.05 was considered significant.

## Supplementary Materials

Supplementary Figures

Supplementary Table

Supplementary Video 1

Supplementary Video 2
