# Supplementary material for: Restoration of aged hematopoietic cells by their young counterparts through instructive microvesicles release
Source: Aging (Albany NY). 2021 Nov 11;13(21):23981–4016. doi: 10.18632/aging.203689 (PMC8610119; doi:10.18632/aging.203689)
Supplement: Supplementary Figures [file aging-13-203689-s001.pdf]

SUPPLEMENTARY FIGURES

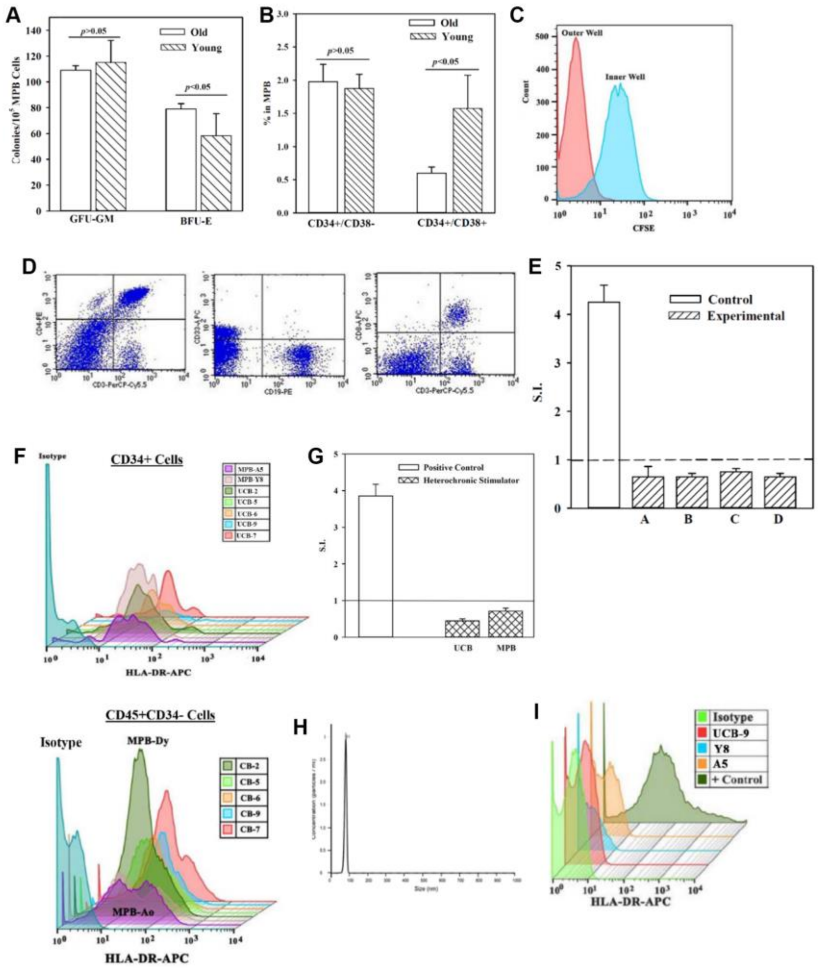

Supplementary Figure 1. (**A**) Clonogenic assay for CFU-GM and BFU-E with MPB cells from young and aged donors (*n* = 8 for each group, each tested in triplicate). The number of CFU-GM/10^5 cells are presented as the mean ± SD. (**B**) Flow cytometry with cells from young and aged donors. CD45+ cells were gated for CD34 and then divided based on CD38. The results are presented as the mean MFI ± SD for four aged and four young MPBs. (**C**) Heterochronic transwell cultures contained CFSE-labeled young MPBs in the inner wells and unlabeled aged MPBs in the outer wells. At weekly intervals up to week 4, the inner wells were examined for CFSE. The histogram represents the result at the 4-wk time point. (**D**) Representative images of flow cytometry for T- (CD3 and CD4), B- (CD19) and myeloid (CD33) cells in the aged MPBs. (**E**) One-way MLR with restore aged MPB as stimulator and naïve autologous aged MPB as responder. The stimulator cells were derived from heterochronic cultures with aged MPBs (arbitrarily labeled A–D), each tested with two different young MPBs in the inner wells. After 4 wks, the restored aged MPBs were γ-irradiated for testing with in the one-way MLR as stimulator cells. The responder cells were viable freshly thawed autologous MPBs. Positive control used unrestored allogeneic MPBs from young or aged donors. The results are presented as mean stimulation indices (S.I.) ± SD for each donor, each tested with two young MPBs in duplicate. The positive controls, which represented 4 experimental points, two with allogeneic aged MPBs and 2 with allogeneic young MPBs showed similar responses and therefore plotted on the same bar. Each MLR test was performed in quadruplicate. (**F**) Flow cytometry for MHC-II using CD45+/CD34+ cells (top panel) and CD34-/CD45+ (bottom panel) from 5 randomly selected UCB and MPBs from a young and an aged donor. The cells were labeled with anti-HLA-DR-APC or isotype. Shown is representative of a young and an aged MPB donor. The results indicated higher density of MHC-II on CD34+ and CD34- UCB as compared to MPB from the aged young donors. (**G**) MLR reactions were repeated for 'E' except that UCB replaced young MPBs in the inner wells. The results are shown as the mean S.I. ± SD from five heterochronic cultures in which four aged MPBs were restored with five different UCB. Each heterochronic culture was done in duplicate and the MLR reactions, in quadruplicates. (**H**) Representative histogram of MVs, isolated from the media of heterochronic cultures, by nanotracker (Nanosight, Malvern, Westborough, MA). (**I**) MVs were captured onto CD63-coupled beads followed by labeling with anti-HLA-DR-APC. The labeled beads were subjected to flow cytometric analyses. Representative results show the results of MVs from heterochronic cultures with UCB in the inner wells. Parallel analyses were performed with media from isochronic cultures containing young and aged MPBs. Positive control (+ control) for the flow cytometric technique used anti-CD3 activated mononuclear cells.

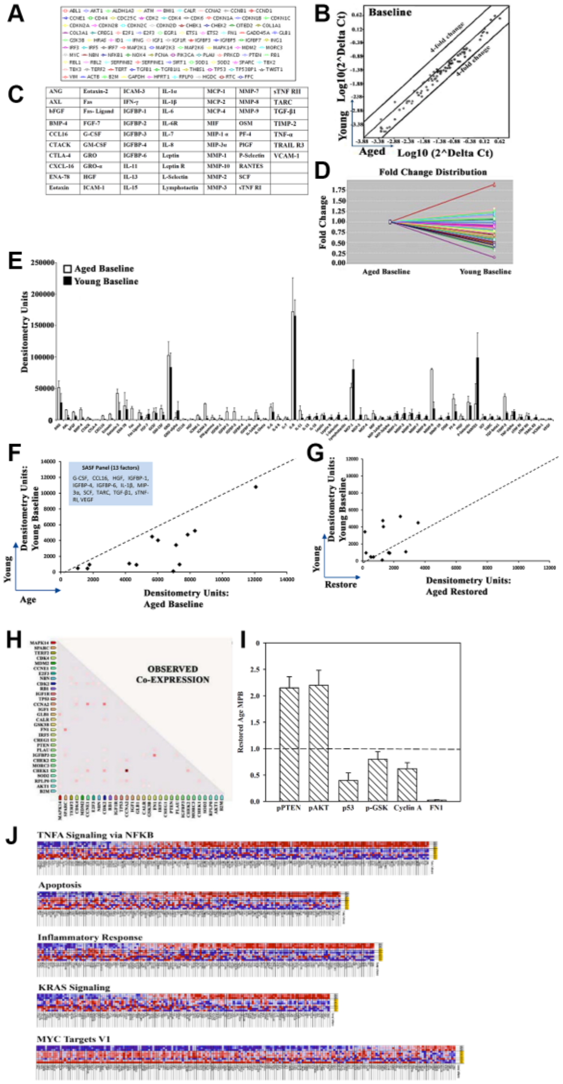

Supplementary Figure 2. (**A**) The 84 age/senescence-related genes in the PCR arrays. (**B**) The results of the analyses in `A' were calculated as follows: ΔC_t between gene-of-interest and housekeeping genes and the values used to calculate fold change. Shown are the boundaries with 4-fold changes in gene expression. (**C**) Custom senescence-associated secretome (SASP) of 64 factors tested for released factors in aged and young bases line as well as restored samples. (**D**) Line plots of 4 biological replicates illustrate the fold change of proteins between aged and young baseline MPB. (**E**) The densitometric results of SASP analyses with media from day 1 heterochronic cultures. Densitometric units were calculated and plotted as a bar graph comparing expression of individual factors among aged and young. Results are presented as the mean ± SD, n = 4. (**F**) The dot plot shows 13 differentially expressed proteins between young and old MPB. The dotted line $y = x$ indicates no change between groups. (**G**) Plots similar to 'F' were made to determine similarities between young and restored aged MPBs. (**H**) The plots shows co-expression of genes in the 30 genes in the boxed region of Figure 1H. The dots represent the association between genes. (**I**) Normalized band densities for Western blots in Figure 1J (n = 3, ± SD). (**J**) The full heat maps of Figure 2D.

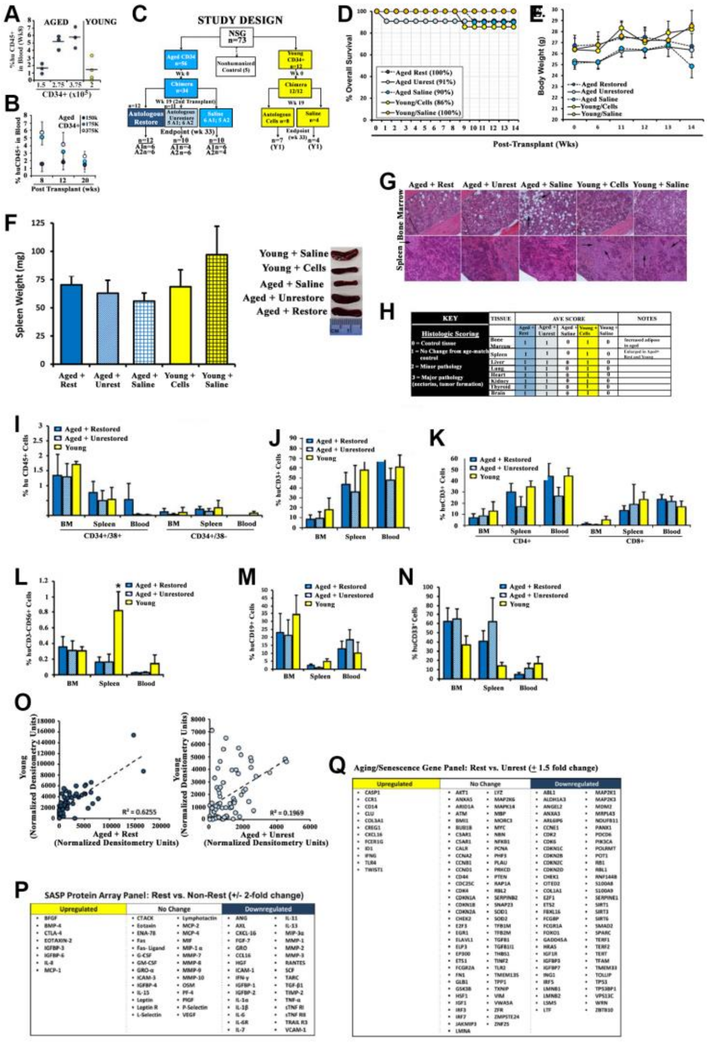

Supplementary Figure 3. Procedural and safety monitoring of huNSG mice. (**A**) Optimize the number of aged CD34+ cells in NSG mice to achieve chimeria. Shown is the engraftment (human chimerism) at wk 8, based on human CD45+ cells in peripheral blood. (**B**) The transplant in 'A' was repeated and chimera assessed up to 20 wks as for 'A'. (**C**) Study design using huNSG to study *in vivo* restoration. A total of 68 irradiated mice were transplanted with aged (n = 56) or young (n = 12) $CD34^+$ cells. 34/56 mice transplanted with aged CD34+ cells successfully achieved chimera, based on the criteria in 'A' and 'B'. 12/12 mice successfully engrafted with young $CD34^+$ cells. Cutoff for enrolling mice in the second transplant with cells from heterochronic or isochronic cultures was set at ≥ 1% $huCD45^+$ cells in blood. Mice displaying 0.5–1% chimera were enrolled in the saline treatment arms, and mice displaying < 0.5% chimerism were not enrolled in the study. The injected cells were from two different aged donors (A1 and A2), restored/heterochronic or unrestored/isochronic (**D**) Kaplan-Meier plot for huNSG survival. (**E**) Body weight at wk 14 following the 2nd transplant with restored MPB (heterochronic cultures) or from isochronic cultures with aged cells. (**F**) Endpoint spleen weights with representative images of the spleen at right. Results are presented as the mean ± SEM. $^*p \le 0.05$ vs. control. Histologic evaluation of tissues from huNSG treatment groups at the study end-points: (**G**) Major organs and immune tissues were harvested and then stained by H&E. Shown are representative sections of femurs (top panels) and spleens (bottom panels) at 10X magnification. (**H**) All harvested tissues were examined for tissue necrosis and tumorigenesis with the treatment groups being compared with age-matched control tissue. The black arrows at the top panel show increased adipocytes in mice femurs given only aged cells whereas similar area of adipocytes was not identified in mice with a second transplant of restored cells. The arrows on the lower panels showed increased hematopoietic activity in the spleen of mice given restored or young cells. Phenotypic analyses for human hematopoietic and immune cells in huNSG mice: Nucleated cells from blood, bone marrow (BM) and spleen were gated for $huCD45^+$. After this, the gated population was analyzed for (**I**) hematopoietic stem ($CD34^+38^-$) and progenitor ($CD34^+38^+$) cells; (**J**) T-cells ($CD3^+$); (**K**) T-helper ($CD4^+$) and cytotoxic ($CD8^+$) cells; (**L**) natural killer cells ($CD3^-56^+$); (**M**) B-cells ($CD19^+$); and (**N**) myeloid cells ($CD33^+$). The results are presented as the mean ± SEM. Senescence and age-related gene and protein expression in huNSG treatment groups: (**O**) Scatterplots comparing senescence-associated secretory factor (SASF) expression in plasma of mice transplanted with either aged restored (left plot) or non-restored (right plot) cells compared to young. Values are normalized by background subtraction of SASF levels in non-humanized control NSG mice. Results are presented as mean densitometry units, with description of upregulated, downregulated and no change in expression SASFs listed in (**P**). (**Q**) List of aging- and senescence-related genes whose expression is upregulated, downregulated or no change in human cells isolated from huNSG BM. Classifications in P and Q are based on a 1.5-fold change cutoff.

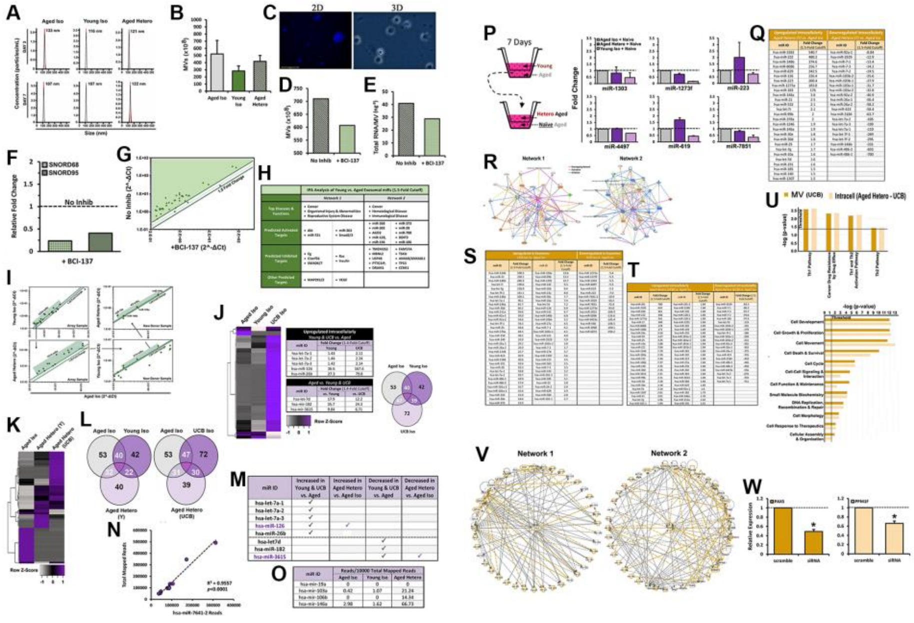

Supplementary Figure 4. MVs and their miRNAs in heterochronic (restored) and isochronic (unrestored) cultures (**A**–**H**). (**A**) Nanoparticle tracking analyses (NTA) of MVs from day 3 and 7 heterochronic cultures. (**B**) MVs in heterochronic or isochronic control cultures were isolated on days 4 and 7, and then pooled. The total number of particles were quantified by NTA, mean ± SD, n = 4. (**C**) CMAC-labeled MVs from 3-day heterochronic cultures were added to naïve aged MPBs. Shown are 2D and 3D images of MVs entering the cells (blue), imaged by EVOS fl and confocal microscopy, respectively. (**D**) Effect of AGO2 inhibitor (BCI-137) on MV release. MVs were collected at days 4 and 7 from the media of heterochronic cultures, which were established in the presence of BCI-137 or vehicle (no Inhibitor). The particles were pooled for quantitation by NTA. The values are presented as the mean ± SD, n = 4. (**E** and **F**) MVs from `D' were quantitated for total RNA (**E**) or small RNA (**F**). (**G**) MVs from `C' were analyzed with miRNA arrays. The results are shown for enrichment of exosomal miRNAs in cultures without inhibitor vs. vehicle in a scatterplot, based on 1.5-fold cutoff. (**H**) Shown are the output of Ingenuity Pathway Analysis (IPA) using commonly expressed miRNAs with differential expression using 1.5-fold cutoff. The data 'F' and the analyses compared young vs. aged isochronic cultures. (**I**) Validation of miFinder qPCR array by individual qPCR experiments in array (left panels) and fresh donor samples (right panels). Gating scheme depicts miRNAs that are upregulated in young isochronic and heterochronic vs. aged isochronic cultures. Results are depicted by scatterplot with 1.25-fold and 1.05-fold cutoffs in array and fresh donor samples, respectively. Array and individual qPCR studies were normalized to RNU6, SNORD68 and SNORD95 and presented as fold change, with a value of 1 representing control. Characterizing the young intracellular miRNAs and ascribing a role for miRNAs in the mechanism of restoration (**J**–**P**). (**J**) Small RNA was purified from aged, young and UCB isochronic cultures for whole miRNA sequencing. All miRNAs exhibiting greater than 100 mappable reads were further analyzed. Differential RNA expression is denoted by heatmap, with miRNA exhibiting greater than 1.4-fold difference among aged vs. UCB and young samples. Outer area of the Venn diagrams depicts total number of intracellular miRNAs with greater than 100 mappable reads in age-matched isochronic samples. Overlapping areas represent common miRNA among samples. (**K**, **L**) Studies, similar to 'I', compared the miRNAs obtained from sequencing of aged isochronic and heterochronic (young-aged, UCB-aged) samples. (**M**) MiR showing differential expression in `J' were compared to miR showing increased or decreased expression in heterochronic (aged-young) vs. aged isochronic cultures in `K' and `L'. The results are tabulated to illustrate candidate miRNAs whose expression patterns are coincident with aged cell restoration. (**N**) Scatterplot depicting linear correlation between exosomal miR-7641-2 expression and total mappable reads, n = 10. (**O**) Expression of early exosomal candidate miRNAs from miFinder array studies in sequencing dataset. Results are shown for isochronic and heterochronic cultures as average reads per 10000 total mapped reads. (**P**) To evaluate whether candidate MV miRNAs can be propagated after aged cell restoration, aged and young cells from 7-day isochronic cultures or aged cells from heterochronic culture were harvested at day 7 and transferred to fresh transwell cultures with naïve aged cells for an additional 7 days. On the 3rd (day 10) and 7th (day 14) day of the propagation culture, MVs were isolated and probed for candidate miRNA expression by qPCR. Results were normalized to miR-7641-2 expression and presented as fold change, with a value of 1 representing control (gray bars). Results are presented as the mean ± SEM, n = 3, unless otherwise noted. $^{*}p \leq 0.05$ vs. control. Identification of potential young MV miRNA targets in aged cells (**Q**–**W**). (**Q**) Up- and downregulated intracellular miRNAs comparing aged heterochronic (aged-young) vs. isochronic cultures with a 1.5-fold cutoff, and their (**R**) predicted activation/inhibition networks after IPA. Up- and downregulated (**S**) MV miRNAs comparing UCB vs. aged isochronic and (**T**) intracellular miRNAs comparing aged heterochronic (aged-UCB) vs. isochronic cultures with a 1.5-fold cutoff. (**U**) Illustration of the top cellular functions (left graph) and canonical pathways (right graph) predicted by (**V**) these networks are shown. (**W**) Validation of siRNA knockdown of target candidates in cells from aged donors. Results were normalized to β-Actin expression and presented as fold change, with a value of 1 representing control (scrambled siRNA). Results are presented as the mean ± SEM, n = 3, unless otherwise noted. $^{*}p \leq 0.05$ vs. control.

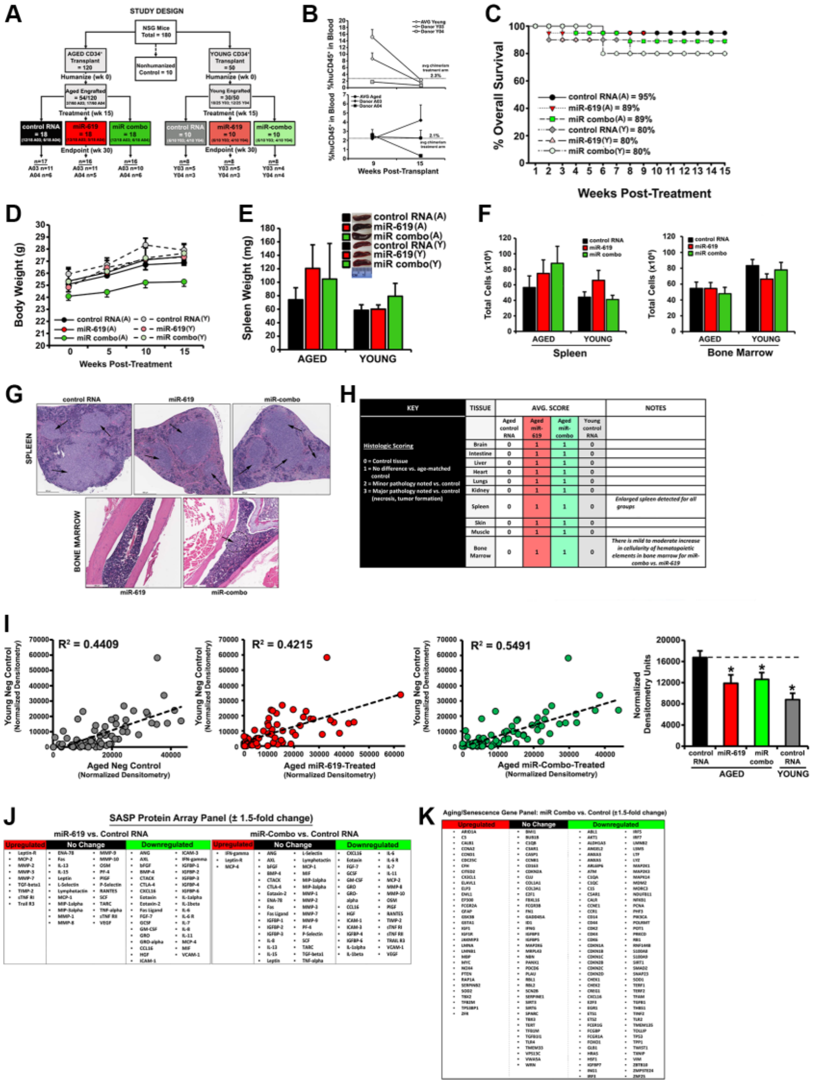

Supplementary Figure 5. Procedural and safety monitoring of humanized mice from with miRNA-mediated restoration. (**A**) Study design with 170 irradiated mice, transplanted with aged (*n* = 120) or young (*n* = 50) CD34⁺ cells, with 54/120 mice successfully engrafted with aged, and 30/50 mice achieving chimera, based on ≥ 1% hucD45⁺ cells in blood. Mice displaying 0.5–1% chimera were enrolled in the saline treatment arms (not shown), and mice displaying < 0.5% chimera were not enrolled in the study. (**B**) Bleeds were performed on mice transplanted with aged (A03 or A04) and young donor (Y03 and Y04) CD34⁺ cells, and chimera evaluated at 9- and 15-weeks post-transplant. Average chimera of the aged (top graph) and young (bottom graph) donors enrolled in the study are shown. (**C**) Kaplan-Meier plot for huNSG overall survival post-treatment and (**D**) mouse body weights for the 15 weeks following the 2nd transplant. Percent survival is displayed in the key (inset). (**E**) Mouse spleen weights at study endpoint, with representative spleen images at the top right of figure. Total (**F**) spleen and (**G**) bone marrow cellularity at study endpoint, mean ± SEM. Histologic evaluation of tissues from huNSG treatment groups: At study endpoint, major organs and immune tissues were harvested. (**G**) H&E staining of mouse spleen (top panels) and bone marrow (bottom panels), 4X magnification. (**H**) All harvested tissues were examined by a pathologist for tissue necrosis and tumorigenesis. Treatment groups were compared to age-matched control tissue for pathological comparison. Senescence- and aging-related gene and protein expression in huNSG treatment groups from expanded study. (**I**) Scatterplots comparing senescence-associated secretory factor (SASF) expression in plasma of mice transplanted with either aged + negative control (gray dot plot), aged + miR-619 (red dot plot) or aged + miR-combo (green dot plot) cells compared to young control. Values are normalized by background subtraction of SASF levels in non-humanized control NSG mice. Results are presented as mean densitometry units, with average total SASF expression among each group also shown for comparison (far right bar graph). Enumeration of SASFs upregulated, downregulated or not changed for miR-619 vs. control (left Table) or miR-combo vs. control (right Table) is listed in '(**J**)'. (**K**) List of aging- and senescence-related genes whose expression is upregulated, downregulated or not changed in human cells isolated from huNSG BM in miR-combo treated mice vs. control. Classifications in 'J' and 'K' are based on a 1.5-fold change cutoff. Results are presented as the mean ± SEM. $^{*}p \leq 0.05$ vs. control.

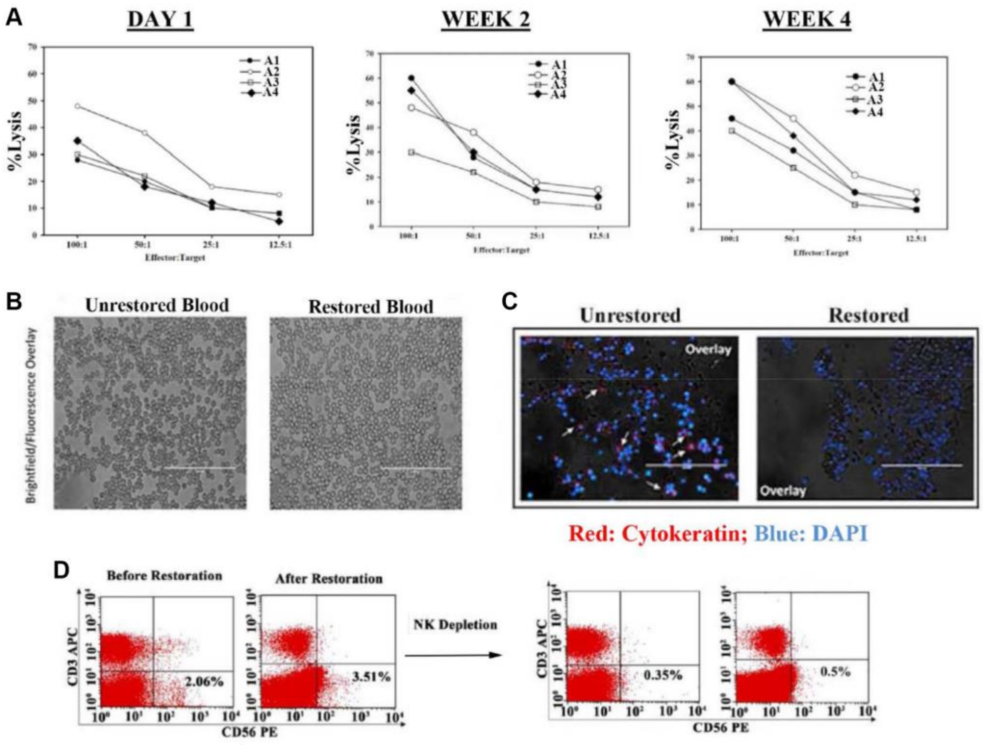

Supplementary Figure 6. (**A**) Timeline natural killer (NK) activity in restored age MPBs. NK activity was performed as described in Materials and Methods at day 1 and, wks 2 and 4 MPBs from heterochronic cultures. The results are shown for four donors, each restored with three different young MPBs. (**B**) Blood smears for GFP+ cells indicated undetectable GFP positivity in mice at 12 months post-transplant. GFP served as a surrogate of cancer stem cells. (**C**) Femurs from mice at 12 months after transplantation with restored or unrestored MPBs. The femurs were dissected longitudinally and then washed with PBS. The cells close to the endosteal regions were scraped and placed onto microscope slides, fixed with 1% paraformaldehyde and then labeled with rabbit anti- human pan cytokeratin (1/1000) for human breast cancer cells and secondary labeling with PE-anti-mouse IgG. DAPI labeling for nuclear labeling of all cells. The slides were immediately imaged with the Evos fl2 auto imager. Representative images show the overlays of the labeled and bright field images at 100x magnification. (**D**) Representative results for CD56+ cells in restored and unrestored MPBs, with or without NK cell depletion.
