# Supplementary material for: Restoration of aged hematopoietic cells by their young counterparts through instructive microvesicles release
Source: Aging (Albany NY). 2021 Nov 11;13(21):23981–4016. doi: 10.18632/aging.203689 (PMC8610119; doi:10.18632/aging.203689)
Supplement: Supplementary Table [file aging-13-203689-s002.pdf]

SUPPLEMENTARY TABLE

Supplementary Table 1. Mobilized Peripheral Blood (MPB) donor demographics.

Donor ID	Age	Sex	Height (in)	Weight (lb)	Ethnicity	TNC Count (10^9)	$CD34^+$ Count (10^6)
				**Old**			
**A1**	61	M	70	162	Caucasian	71.9	90.0
**A2**	60	M	70	190	Caucasian	43.6	185
**A3**	61	M	68	149	Caucasian	32.2	46.8
**A4**	61	M	67	172	Caucasian	47.0	93.9
**A5**	74	M	NR	NR	Caucasian	*	
**A6**	66	M	NR	NR	Caucasian	*	
**A7**	68	M	NR	NR	Caucasian	*	
**A8**	70	M	NR	NR	Caucasian	*	
				**Young**			
**Y1**	28	M	67	140	African American	42.2	354
**Y2**	22	M	66	146	Hispanic	79.2	192
**Y3**	20	M	67	160	Caucasian	45.5	200
**Y4**	20	F	60	120	Hispanic	43.2	82.2
**Y5**	21	M	69	130	Hispanic	20.1	47.1
**Y6**	28	M	NR	138	Caucasian	*	
**Y7**	30	F	NR	128	Caucasian	*	
**Y8**	29	M	NR	130	Caucasian	*	

Abbreviation: NR: Not recorded. *Aliquots (~15–25 mL) of mobilized peripheral blood donated for research.

Demographics of Umbilical Cord Blood (UCB)

ID	UCB Volume (mL)	Time (h): Delivery to Process
UCB1	68	25
UCB2	56	8
UCB3	75	22
UCB4	71	24
UCB5	46	10
UCB6	67	21
UCB7	61	22
UCB8	108	25
UCB9	59	25
UCB10	73	20

Supplementary Video 1. Video showing entry of MV into cells.

Supplementary Video 2. Video showing MV entering cells.
